# PM-DUnet: Fusing long-range dependencies and attention in a dual-U architecture for thyroid nodule segmentation

**DOI:** 10.1371/journal.pone.0353684

**Published:** 2026-07-30

**Authors:** Shaoqiang Wang, Linhao Zhang, Guiling Shi, Zhongran Liu, Yuanyuan Zhang, Tiyao Liu, Yawu Zhao, Yuchen Wang, Xiaochun Cheng

**Affiliations:** 1 Qingdao University of Technology, Qingdao, Shandong, China; 2 China University of Petroleum (East China), Qingdao, China; 3 School of Medical Informational Engineering, Shandong University of Traditional Chinese Medicine, Jinan, Shandong, China; 4 Swansea University, Swansea, Wales, United Kingdom; University of Electronic Science and Technology of China, CHINA

## Abstract

For medical image segmentation, accurately balancing local details and global long-range dependencies is critical to tackling thyroid nodule challenges (variable sizes, ambiguous boundaries, complex context). Traditional CNNs excel at local feature extraction but are constrained by local receptive fields, hindering efficient global dependency modeling. To address this, we propose a Parallel Mamba Dual-U Network (PM-DUNet). It adopts a cascaded dual U-Net encoder-decoder for two-stage “coarse-to-fine” segmentation refinement. We design a Multi-Path Parallel Mamba (MPM) module—using State Space Models (SSMs)—to efficiently model global context with linear complexity. Additionally, Squeeze-Excitation Downsampling (SED) and Spatial Attention Upsampling (SAU) modules are integrated to adaptively enhance key features in encoding/decoding. Results show PM-DUNet achieves highly competitive performance and outperforms state-of-the-art methods on most core metrics, verifying its effectiveness and robustness for complex medical image segmentation. Our code is available on https://github.com/Andrevict/MPDUNet.

## Introduction

Thyroid cancer ranks among the most prevalent endocrine malignancies globally, with its incidence showing a consistent upward trend in recent years. Ultrasound (US) imaging has become the primary screening modality for the detection and diagnosis of thyroid nodules, owing to its non-invasive, real-time, and cost-effective nature. In clinical practice, the accurate segmentation of thyroid nodules from US images is paramount for subsequent feature analysis, differentiation between benign and malignant lesions, and the formulation of treatment plans. However, traditional clinical diagnosis heavily relies on manual delineation by radiologists. This process is not only laborious and time-consuming but also susceptible to significant inter-observer variability due to subjective experience and fatigue [1–3]. In recent years, with the rapid advancement of artificial intelligence, automated medical image segmentation methods based on deep learning have made remarkable progress, demonstrating immense potential in the task of thyroid nodule segmentation. CNNs, epitomized by U-Net and its numerous variants, have become the dominant technology in this field due to their powerful local feature extraction capabilities. Limitations still face considerable challenges when processing complex thyroid US images. Thyroid nodules often exhibit high variability in size and shape. They are frequently accompanied by ambiguous boundaries, low contrast, and speckle noise, all of which significantly increase the difficulty of precise segmentation. More critically, a fundamental limitation of traditional CNNs lies in their inherent local receptive fields, which restrict their ability to capture global contextual information and long-range dependencies effectively. This capability is crucial for accurately identifying and distinguishing nodules with ill-defined borders from the complex surrounding normal tissue. Although some studies have attempted to incorporate Transformer-based architectures to address this issue, their quadratic computational complexity often leads to a large number of parameters, requiring extensive training data and computational resources, which are typically scarce in the medical imaging domain. We review three main research directions in the field of medical image segmentation: methods based on CNNs, the application of attention mechanisms, and emerging architectures for long-range dependency modeling.

**CNN-based medical image segmentation.** Since the rise of deep learning, CNNs have become the mainstream approach for medical image segmentation tasks. Among them, the U-Net [4] architecture proposed by Ronneberger et al. is a landmark achievement. U-Net’s innovative encoder-decoder structure, featuring symmetric downsampling and upsampling paths, utilizes skip connections to merge shallow, high-resolution features from the encoder with deep, semantic features from the decoder. This design effectively addresses the common issue of detail loss in segmentation tasks. Even today, optimized CNN variants like nnU-Net [5] remain competitive in medical image segmentation by adapting network configurations to dataset characteristics, highlighting the enduring value of CNN-based frameworks. Building on the outstanding performance of U-Net, a plethora of variant models have been proposed. For instance, U-Net++ [6] introduces dense skip-connection pathways to enhance feature fusion across different levels further, effectively mitigating the semantic gap between the encoder and decoder. ResU-Net [7] integrates residual learning units into the U-Net framework, enabling the construction of deeper networks to improve feature extraction capabilities while avoiding the vanishing gradient problem. Furthermore, some studies have designed cascaded or dual-U-Net structures, such as DoubleU-Net [8], to perform a secondary refinement of the initial segmentation results, thereby achieving more precise outcomes. However, CNNs inherently rely on local receptive fields, which limit their ability to capture global contextual information—a critical shortcoming when handling low-contrast medical images with ambiguous boundaries, such as thyroid nodules in ultrasound imaging. Recent benchmarks also indicate that while CNNs excel at local feature extraction, they often struggle with long-range dependency modeling compared to transformer-based architectures in complex anatomical scenarios [9].

**Attention mechanisms in segmentation.** To compensate for the limited receptive fields of CNNs, attention mechanisms have been widely incorporated into segmentation networks. The core idea is to enable the model to focus on the most informative feature regions adaptively. Attention mechanisms [10] can be broadly categorized into channel attention, spatial attention, and their combinations. A representative of channel attention is the Squeeze-and-Excitation block [11], which explicitly models the interdependencies between channels to learn a weight for each channel, thereby enhancing crucial feature channels while suppressing less important ones. Spatial attention, on the other hand, aims to identify “where” is important by learning a spatial weight map to emphasize feature responses in key areas. AttU-Net [12] is a classic example that integrates a spatial attention module into the skip connections of U-Net, allowing the model to suppress irrelevant activations in background regions before feature fusion. Our work also leverages the advantages of both mechanisms by integrating channel and spatial attention into our downsampling and upsampling modules, respectively, to achieve more efficient information filtering during feature scale transformation.

**Long-range dependency modeling.** Effectively modeling long-range dependencies is crucial for understanding global context, especially when dealing with large lesions or complex anatomical structures. To break through the limitations of CNNs, some researchers began to explore Transformer architectures based on self-attention mechanisms [13]. TransUNet [9] was a pioneering work that employed a Transformer as the encoder to extract global features and combined it with a CNN-based decoder, achieving significant success in medical image segmentation. However, the self-attention mechanism in the standard Transformer has a quadratic computational and memory complexity with respect to the input sequence length, which limits its application in high-resolution images. To address this issue, various efficient Transformer variants have been proposed [14,15]. Nevertheless, the high computational cost remains a challenge. Recently, State Space Models(SSMs) [16], particularly Mamba [17], have garnered widespread attention as an emerging paradigm for sequence modeling. Mamba, through its selective state-space mechanism, can efficiently capture long-range dependencies with linear computational complexity. Inspired by this, our work innovatively designs the MPM module, aiming to combine the global modeling capability of SSMs with the local feature extraction strengths of CNNs to enhance the model’s contextual awareness more efficiently. To tackle the aforementioned challenges, we propose a novel **PM-DUNet**. Our network employs an innovative dual U-Net cascaded encoder-decoder architecture, which progressively refines segmentation accuracy through a two-stage “coarse-to-fine” strategy [18]. To overcome the limitations of traditional convolutions, we introduce the **MPM module**, which leverages advanced SSMs to model global dependencies with linear complexity efficiently. Furthermore, we have proposed novel **SED module** and **SAU module**, respectively, to enhance the representation of critical information and suppress irrelevant noise during feature encoding and decoding. Through the synergistic effect of these designs, our model strikes an excellent balance between accuracy and robustness. The main contributions of this paper can be summarized as follows:

- We propose a novel PM-DUNet, which utilizes a dual U-Net cascade architecture to achieve precise segmentation of thyroid nodules through a two-stage process of feature learning and refinement.
- We innovatively propose the MPM module into the skip connections to efficiently capture global contextual information with linear computational complexity, enhancing the model’s ability to handle long-range dependencies.
- We proposed attention-enhanced SED and SAU modules that adaptively focus on salient channel and spatial information during feature scale transformation, thereby reducing information loss.

## Materials and methods

### Materials

To comprehensively evaluate the performance and generalization capability of our proposed model, we conducted experiments on three publicly available thyroid ultrasound image datasets: DDTI, TG3K, and TN3K. These datasets encompass thyroid nodule images from various sources with different annotation styles, providing a solid foundation for verifying the robustness of our model.

**DDTI dataset [**19**]:** This dataset contains thyroid ultrasound images collected between 2010 and 2016, which were retrospectively analyzed by experienced radiologists. It includes 789 thyroid nodule images from 535 patients, with each image accompanied by a precise pixel-level segmentation mask. These images capture a wide diversity of nodules in terms of morphology, size, and texture, posing significant challenges for segmentation algorithms.

**TG3K dataset: [**20**]** This is a large-scale, high-quality dataset for thyroid nodule segmentation, comprising 3585 ultrasound images extracted from 16 ultrasound video sequences. The images in this dataset were acquired using various ultrasound devices, thus exhibiting high diversity in imaging style and quality. All images were annotated by experts with over five years of experience. The TG3K dataset offers a valuable resource for training and evaluating segmentation models that can perform consistently across images from different equipment sources.

**TN3K dataset: [**21**]** This dataset consists of 3493 thyroid ultrasound images from 2421 patients, focusing on the fine-grained segmentation of thyroid nodules. Similar to TG3K, the images in this dataset originate from multiple hospitals and a variety of devices, ensuring data diversity. The establishment of this dataset aims to promote the development of more refined and accurate automated thyroid nodule segmentation techniques.

For all the datasets mentioned above, we followed the standard practice in the literature by randomly splitting them into training,validation, and testing sets at an 8:1:1 ratio at the patient level. All images were resized to 224×224 pixels and normalized before being fed into the model. Specifically, the division is performed based on the patient dimension to ensure that all images belonging to the same patient are assigned exclusively to the same set, preventing cross-set distribution and ensuring no data leakage occurs.

### Method

#### Dual-U model architecture.

**Fig 1:**
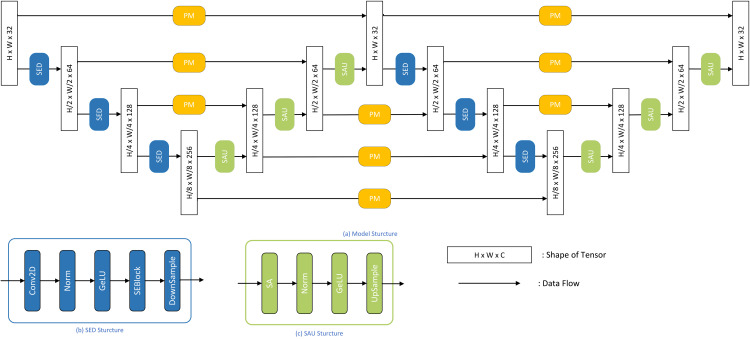
PM-DUNet. The overall architecture of our proposed PM-DUNet. The network consists of a cascaded dual U-Net encoder-decoder structure, featuring two main stages: a primary encoder-decoder for coarse segmentation and a secondary one for refinement. The encoder paths are composed of the SED module, while the decoder paths utilize the SAU module. Our proposed MPM module is embedded within the skip connections to capture global context efficiently.

In the field of medical image segmentation, accurately capturing both local detailed features and global long-range dependencies is crucial to address challenges such as variable sizes of lesion areas, blurred boundaries, and complex contextual information. Traditional CNNs excel at extracting local features, but they are limited by their inherent local receptive fields, making it difficult to efficiently model global dependencies. To overcome these limitations, we have designed and proposed a novel medical image segmentation network, which we name the Parallel Mamba Dual-U Network. The core idea of PM-DUNet lies in constructing a dual U-shaped encoding and decoding structure. Through a two-stage process of feature extraction and reconstruction, it achieves both initial segmentation of the target features and subsequent refinement and correction. As shown in Fig 1, this framework integrates three key modules that we have meticulously designed:

#### Multi-Path Parallel Mamba module.

**Fig 2:**
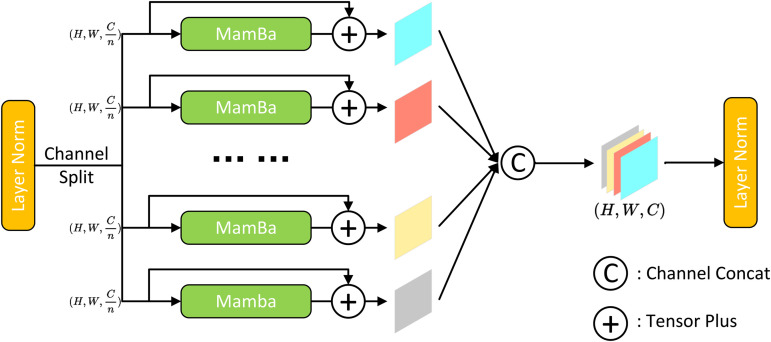
MPM module. Detailed structure of our proposed MPM module. This module is designed to capture global contextual information efficiently. The input features first undergo Layer Normalization and are then split into n parallel paths along the channel dimension.

To effectively capture long-range dependencies across the entire feature map in medical images, we designed the MPM module, as illustrated in Fig 2. Traditional CNNs are limited by their local receptive fields, making it difficult to efficiently model global context. While the self-attention mechanism can address this issue, its computational complexity grows quadratically with the increase in spatial resolution. The MPM module, by introducing the efficient SSM Mamba and parallelizing it along the channel dimension, aims to achieve powerful global context modeling capabilities with linear computational complexity. It is particularly well-suited for the skip connection paths in a U-Net architecture to enhance feature representation.

Given an input feature map $X\in {\mathbb{R}}^{B\times C_{in}\times H\times W}$ from a specific layer of the encoder or decoder, where *B* is the batch size, $C_{in}$ is the number of input channels, and *H* and *W* are the height and width of the feature map, the processing flow of the MPM module is as follows:

**Feature serialization.** The input 2D spatial feature map, $X\in {\mathbb{R}}^{B\times C_{\text{in}}\times H\times W}$, is first serialized into a 1D token sequence to align with Mamba’s operational requirements for sequential data. This is achieved by flattening the spatial dimensions (*H*, *W*) into a sequence of length N=H×W, which results in a reshaped tensor $X_{\text{seq}}\in {\mathbb{R}}^{B\times N\times C_{\text{in}}}$.

**Normalization and channel parallelization.** For training stability and to facilitate diverse feature learning, the serialized tensor *X*_seq_ first undergoes Layer Normalization. Subsequently, the normalized tensor is partitioned along its channel dimension into four distinct subsequences:


$$[x_{1},x_{2},x_{3},x_{4}]={\text{Split}}_{c}(\text{LayerNorm}(X_{\text{seq}})),$$ (1)

where each subsequence $x_{k}\in {\mathbb{R}}^{B\times N\times (C_{\text{in}}/4)}$, and ${\text{Split}}_{c}(\cdot )$ denotes the channel-wise splitting operation.

**Parallel Mamba processing.** Each subsequence $x_{k}$ is then processed independently by a weight-shared Mamba block. Leveraging its selective state-space mechanism, Mamba efficiently captures long-range dependencies within each sequence. To preserve the original feature information and enhance gradient flow, we incorporate a learnable scaled residual connection:


$$\begin{array}{c} y_{k}=M(x_{k})+\alpha \cdot x_{k},\;\text{for }k\in \{1,2,3,4\}. \end{array}$$ (2)

Here, M(·) represents the core Mamba operation, and α is a learnable scalar parameter (denoted as skip_scale) shared across all parallel paths.

**Feature fusion and projection.** Following parallel processing, the four output sequences $\{y_{k}{\}}_{k=1}^{4}$ are concatenated along the channel dimension, fusing the contextual information learned from the different channel subspaces. The resulting merged sequence is normalized and then transformed by a linear layer to project the feature dimension from *C*_in_ to the target output dimension *C*_out_:


$$\begin{array}{c} Y_{\text{proj}}=\text{Linear}(\text{LayerNorm}([y_{1};y_{2};y_{3};y_{4}])), \end{array}$$ (3)

where [;] signifies concatenation along the channel dimension, yielding the output $Y_{\text{proj}}\in {\mathbb{R}}^{B\times N\times C_{\text{out}}}$.

**Feature reconstruction.** Finally, the processed sequence *Y*_proj_ is de-serialized by reshaping it back into a 2D spatial layout. This step reconstructs the feature map, producing the final module output $Y\in {\mathbb{R}}^{B\times C_{\text{out}}\times H\times W}$. This architectural design allows the MPM module to model global contextual dependencies efficiently. The channel parallelization strategy enriches the feature representation while maintaining computational tractability, rendering MPM a potent and scalable component for deep neural networks.

#### Squeeze-and-Excitation downsampling module.

**Fig 3:**
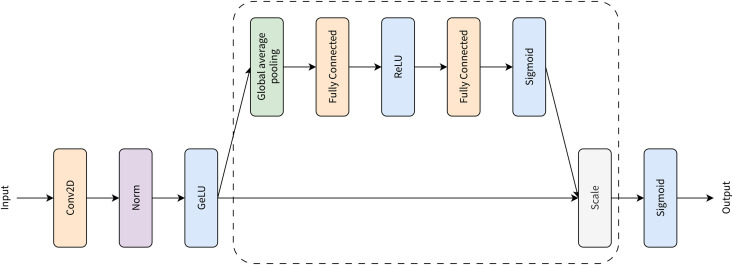
SED module. The architecture of our proposed SED module. The SED module integrates SE channel attention after a standard convolutional block to enhance key feature channels.

Within the encoder path, we employ the SED module to progressively reduce the spatial resolution of feature maps while extracting high-level semantic information. This module integrates a channel attention mechanism after a standard convolutional block, enabling adaptive recalibration of channel-wise feature responses to preserve the most salient information during downsampling. The module structure is shown in Fig 3.

Given an input feature map $X\in {\mathbb{R}}^{B\times C\times H\times W}$, the SED module’s workflow comprises the following three steps:

**Feature transformation.** The input features initially undergo a non-linear transformation via a standard convolutional block. This process is formulated as follows:


$$\begin{array}{c} X^{\prime }=𝒢(\text{GN}({\text{Conv}}_{3\times 3}(X))), \end{array}$$ (4)

where ${\text{Conv}}_{3\times 3}$ denotes a convolution layer with a 3×3 kernel, GN is Group Normalization, and 𝒢 represents the GELU activation function [22]. The output feature map is $X^{\prime }\in {\mathbb{R}}^{B\times C^{\prime }\times H\times W}$.

**Channel attention.** Next, the Squeeze-and-Excitation attention mechanism is applied to the transformed feature map $X^{\prime }$ to learn the importance weight of each channel.

**Squeeze.** Global Average Pooling (GAP) aggregates spatial information into a channel descriptor $z\in {\mathbb{R}}^{C^{\prime }}$. This is achieved by shrinking the feature map $X^{\prime }$ across its spatial dimensions (H×W):


$$\begin{array}{c} z_{c}=F_{sq}(X_{c}^{\prime })=\frac{1}{H\times W}\sum_{i=1}^{H}\sum_{j=1}^{W}X_{c}^{\prime }(i,j). \end{array}$$ (5)

**Excitation.** A bottleneck architecture with two fully-connected (FC) layers [23] models inter-channel dependencies to generate channel-wise attention weights $s\in {\mathbb{R}}^{C^{\prime }}$:


$$\begin{array}{c} s=F_{ex}(z,𝐖)=\sigma ({𝐖}_{2}\delta ({𝐖}_{1}z)), \end{array}$$ (6)

where σ and δ represent the Sigmoid and ReLU activation functions, respectively. ${𝐖}_{1}\in {\mathbb{R}}^{\frac{C^{\prime }}{r}\times C^{\prime }}$ and ${𝐖}_{2}\in {\mathbb{R}}^{C^{\prime }\times \frac{C^{\prime }}{r}}$ are the weights of the two FC layers, and *r* is the reduction ratio.

**Reweight.** The learned weights *s* are applied to recalibrate the feature map $X^{\prime }$, producing the output $X_{se}$:


$$\begin{array}{c} X_{se}=F_{scale}(X^{\prime },s)=s⊙X^{\prime }, \end{array}$$ (7)

where ⊙ denotes channel-wise multiplication, broadcasting each weight $s_{c}$ across the spatial dimensions of the corresponding channel $X_{c}^{\prime }$.

**Spatial downsampling.** Finally, a max-pooling layer with a 2×2 kernel and a stride of 2 is applied to downsample the spatial dimensions, yielding the final output $Y\in {\mathbb{R}}^{B\times C^{\prime }\times \frac{H}{2}\times \frac{W}{2}}$:


$$\begin{array}{c} Y={\text{MaxPool}}_{2\times 2}(X_{se}). \end{array}$$ (8)

#### Spatial attention upsampling module.

**Fig 4:**
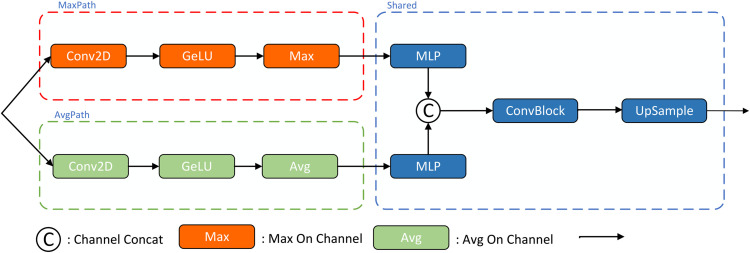
SAU module. The architecture of our proposed SAU module. The module processes features through parallel average-pooling and max-pooling paths followed by a 7×7 convolutional layer.

In the decoder path, we introduce the SAU module, designed to restore spatial resolution and reconstruct fine-grained image details. This module initiates its process with a spatial attention mechanism, enabling the network to focus on critical spatial regions, thereby enhancing the precision of boundary segmentation. The module structure is shown in Fig 4.

Given a deep-level feature map $X\in {\mathbb{R}}^{B\times C\times H\times W}$ from a preceding decoder stage, the detailed operations of the SAU module are as follows:

**Spatial attention.** The module first computes a spatial attention map to identify and emphasize salient spatial locations.

**Channel aggregation.** Channel information is first aggregated across all channels for each spatial position by applying both average-pooling and max-pooling operations in parallel. This produces two distinct spatial descriptors, $F_{avg},F_{max}\in {\mathbb{R}}^{B\times 1\times H\times W}$.

**Attention map generation.** The two descriptors are concatenated and then processed by a convolutional layer with a large 7×7 kernel ($f_{7\times 7}$) to capture spatial dependencies effectively. A Sigmoid function is applied to the result to produce the final spatial attention map $M_{s}\in {\mathbb{R}}^{B\times 1\times H\times W}$:


$$\begin{array}{c} M_{s}=\sigma (f_{7\times 7}([{\text{AvgPool}}_{c}(X);{\text{MaxPool}}_{c}(X)])), \end{array}$$ (9)

where [;] denotes concatenation along the channel axis.

**Feature recalibration.** The resulting attention map $M_{s}$ is applied to the original input feature map *X* via element-wise multiplication to yield a spatially refined feature map $X_{sa}$:


$$\begin{array}{c} X_{sa}=M_{s}⊙X. \end{array}$$ (10)

**Feature transformation and fusion.** The spatially-enhanced feature map $X_{sa}$ then undergoes a series of transformations to enrich its feature representation. First, $X_{sa}$ is passed through a standard convolutional block to obtain a transformed feature map *X*_conv_:


$$\begin{array}{c} X_{\text{conv}}=𝒢(\text{GN}({\text{Conv}}_{3\times 3}(X_{sa}))). \end{array}$$ (11)

Subsequently, to create a more comprehensive representation, the transformed features *X*_conv_ are concatenated with the pre-transformation features $X_{sa}$ along the channel dimension:


$$\begin{array}{c} X_{\text{cat}}=[X_{\text{conv}};X_{sa}]. \end{array}$$ (12)

**Spatial upsampling.** Finally, a transposed convolution with a 2×2 kernel and a stride of 2 is employed to upsample the fused feature map *X*_cat_, doubling its spatial resolution. This produces the final module output $Y\in {\mathbb{R}}^{B\times C_{out}\times 2H\times 2W}$:


$$\begin{array}{c} Y={\text{ConvTranspose}}_{2\times 2}(X_{\text{cat}}). \end{array}$$ (13)

## Results

To validate the effectiveness of our proposed PM-DUNet, we compared it with several existing SOTA segmentation methods, including the classic U-Net [4], U-Net++ [6], and other recent models such as AttU-Net [12], CE-Net [24], UKAN [25], Unext [26], DSU-Net [27], WRANet [28] and TransUnet [9]. The quantitative comparison results for all models are presented in Table 1. We used the TN3K, TG3K, and DDTI datasets with all images resized to 224×224 pixels. The datasets were partitioned into a training set (80%) and a test set (20%) at the patient level to prevent data leakage. The datasets were loaded using data loaders with a batch size of 16 for the training set and 8 for the validation set. All experiments were conducted on an RTX4090 with CUDA 12.8. For optimization, we utilized the Adam optimizer with an initial learning rate of 0.0001 and a weight decay of 0.00001. We chose Dice loss and BCE loss as our loss functions. The training was carried out for a maximum of 200 epochs. To prevent overfitting and ensure reproducibility, an early stopping mechanism was adopted based on the validation loss, with a patience of 15 epochs.

**Table 1:** Quantitative comparison of different models on DDT1, TG3K, and TN3K datasets. The best results are highlighted in bold.

Model	Dice	IoU	Precision	SE
**DDT1 Dataset**				
U-Net(2015)	74.85 ± 0.32	62.28 ± 0.19	81.65 ± 0.47	74.71 ± 0.26
U-Net++(2018)	75.12 ± 0.18	63.52 ± 0.45	80.87 ± 0.31	75.31 ± 0.29
AttU-Net(2021)	75.72 ± 0.42	63.55 ± 0.36	81.95 ± 0.14	77.68 ± 0.38
CE-Net(2019)	72.88 ± 0.25	60.25 ± 0.39	77.15 ± 0.49	75.73 ± 0.17
DSU-Net(2025)	75.31 ± 0.34	62.83 ± 0.21	77.75 ± 0.43	78.25 ± 0.30
TransUNet(2021)	74.23 ± 0.19	62.51 ± 0.44	81.17 ± 0.23	75.92 ± 0.41
UKAN(2024)	76.78 ± 0.38	64.39 ± 0.28	82.65 ± 0.35	80.08 ± 0.46
UNext(2022)	69.21 ± 0.49	55.68 ± 0.31	80.25 ± 0.24	78.15 ± 0.33
WRANet(2023)	76.08 ± 0.22	63.89 ± 0.37	81.61 ± 0.45	76.48 ± 0.16
**Ours**	**77.45 ± 0.30**	**65.11 ± 0.46**	**83.28 ± 0.27**	**80.92 ± 0.35**
**TG3K Dataset**				
U-Net(2015)	96.25 ± 0.15	95.15 ± 0.43	95.98 ± 0.28	96.38 ± 0.37
U-Net++(2018)	97.58 ± 0.34	95.29 ± 0.22	97.19 ± 0.48	97.81 ± 0.16
AttU-Net(2021)	97.71 ± 0.27	95.85 ± 0.41	97.12 ± 0.32	97.55 ± 0.23
CE-Net(2019)	97.15 ± 0.38	95.32 ± 0.26	96.81 ± 0.35	98.11 ± 0.42
DSU-Net(2025)	96.49 ± 0.47	93.95 ± 0.16	96.53 ± 0.29	97.09 ± 0.36
TransUNet(2021)	96.68 ± 0.24	93.38 ± 0.37	96.25 ± 0.40	96.82 ± 0.22
UKAN(2024)	97.88 ± 0.17	95.61 ± 0.42	97.38 ± 0.25	98.29 ± 0.39
UNext(2022)	97.33 ± 0.30	95.03 ± 0.48	96.79 ± 0.19	97.77 ± 0.27
WRANet(2023)	97.79 ± 0.35	95.57 ± 0.23	97.05 ± 0.44	98.33 ± 0.31
**Ours**	**98.02 ± 0.21**	**96.01 ± 0.38**	**97.59 ± 0.43**	**98.45 ± 0.28**
**TN3K Dataset**				
U-Net(2015)	77.22 ± 0.41	67.51 ± 0.17	80.85 ± 0.35	81.69 ± 0.24
U-Net++(2018)	76.75 ± 0.39	66.19 ± 0.25	77.92 ± 0.44	81.85 ± 0.33
AttU-Net(2021)	77.58 ± 0.19	67.23 ± 0.46	80.49 ± 0.21	82.81 ± 0.37
CE-Net(2019)	78.09 ± 0.28	67.88 ± 0.33	81.11 ± 0.46	82.39 ± 0.18
DSU-Net(2025)	76.28 ± 0.45	65.74 ± 0.27	76.95 ± 0.31	81.41 ± 0.48
TransUNet(2021)	77.06 ± 0.33	67.05 ± 0.15	80.28 ± 0.49	83.35 ± 0.32
UKAN(2024)	74.98 ± 0.26	64.27 ± 0.34	76.43 ± 0.41	81.93 ± 0.20
UNext(2022)	71.49 ± 0.36	59.88 ± 0.40	76.05 ± 0.38	77.88 ± 0.34
WRANet(2023)	78.28 ± 0.42	68.15 ± 0.29	80.39 ± 0.20	**83.85 ± 0.47**
**Ours**	**79.28 ± 0.32**	**69.34 ± 0.41**	**82.35 ± 0.36**	83.01 ± 0.19

Table notes: 1) Abbreviations: SE = Sensitivity, IoU = Intersection over Union; 2) All metrics are presented as percentages (%); 3) “Ours” refers to the proposed PM-DUNet.

To thoroughly validate the effectiveness of our proposed components, we conducted a series of ablation studies. These experiments were designed to systematically evaluate the contribution of each key component to the overall performance of PM-DUNet. We investigated two main aspects: (1) the individual contributions of the dual-U structure, the SED and SAU module, and the MPM module; and (2) the impact of varying the number of parallel paths within the MPM module. All experiments were conducted under identical settings to ensure fair comparisons. To isolate the contribution of each component, we started with a baseline U-Net and progressively integrated our proposed modules. The configurations are as follows:

- **Baseline:** A standard U-Net architecture.
- **Baseline + Dual-U:** The baseline model augmented with our cascaded dual U-shaped structure, but without any attention or MPM modules.
- **Baseline + Dual-U + Attn:** The dual-path architecture is further enhanced with the SED and SAU attention modules in the downsampling and upsampling blocks, respectively.
- **PM-DUNet (Full Model):** Our complete proposed model, which includes the dual-path structure, attention modules, and the MPM modules in the skip connections.

The quantitative results are presented in Table 2. The findings clearly demonstrate that each component progressively improves segmentation performance. The introduction of the **Dual-U** structure provides a significant boost over the baseline, confirming that the progressive refinement strategy is effective. Adding the **Attention (Attn)** modules further enhances the results, indicating that adaptively recalibrating channel and spatial features helps the model focus on relevant regions. Finally, the **Full Model**, with the inclusion of the **MPM** modules, achieves the best performance across all datasets and metrics. This underscores the critical role of the MPM module in capturing long-range dependencies, which is essential for accurately segmenting nodules of varying sizes and shapes.

**Table 2:** Component ablation study of our proposed PM-DUNet. The best results are highlighted in bold.

Model	Dice	IoU	Precision	SE
**DDT1 Dataset**				
Baseline (U-Net)	74.91 ± 0.27	62.35 ± 0.19	80.52 ± 0.23	74.82 ± 0.15
Baseline + Dual-U	75.83 ± 0.14	63.71 ± 0.22	81.68 ± 0.28	77.95 ± 0.16
Baseline + Dual-U + Attn	76.95 ± 0.21	64.62 ± 0.18	82.35 ± 0.26	80.23 ± 0.24
**PM-DUNet (Full)**	**77.45 ± 0.23**	**65.11 ± 0.28**	**83.28 ± 0.19**	**80.92 ± 0.22**
**TG3K Dataset**				
Baseline (U-Net)	96.31 ± 0.21	95.21 ± 0.28	95.86 ± 0.17	96.45 ± 0.24
Baseline + Dual-U	97.59 ± 0.25	95.29 ± 0.11	97.12 ± 0.27	97.71 ± 0.19
Baseline + Dual-U + Attn	97.88 ± 0.15	95.51 ± 0.29	97.22 ± 0.12	98.31 ± 0.20
**PM-DUNet (Full)**	**98.02 ± 0.17**	**96.01 ± 0.24**	**97.59 ± 0.21**	**98.45 ± 0.16**
**TN3K Dataset**				
Baseline (U-Net)	77.31 ± 0.18	67.62 ± 0.29	78.12 ± 0.13	81.75 ± 0.26
Baseline + Dual-U	78.15 ± 0.24	67.99 ± 0.20	79.25 ± 0.17	82.58 ± 0.23
Baseline + Dual-U + Attn	78.89 ± 0.27	68.85 ± 0.14	81.63 ± 0.25	82.95 ± 0.11
**PM-DUNet (Full)**	**79.28 ± 0.29**	**69.34 ± 0.18**	**82.35 ± 0.20**	**83.01 ± 0.25**

Table notes: 1) Abbreviations: SE = Sensitivity, IoU = Intersection over Union, Attn = Attention modules (SED + SAU); 2) All metrics are presented as percentages (%).

The core of our MPM module is its multi-path parallel processing strategy. To determine the optimal number of parallel paths, we experimented with three configurations: 2 paths, 4 paths (ours), and 8 paths. The results, shown in Table 3, reveal an interesting trend. Increasing the number of paths from 2 to 4 yields a consistent performance improvement across all datasets. This suggests that a greater degree of parallelism allows the model to capture a more diverse set of long-range dependencies from different channel subspaces. However, further increasing the paths to 8 results in a slight performance degradation. This performance drop is attributed to the reduced channel capacity within the weight-shared Mamba block for each path. Specifically, since the parameters are shared across all parallel paths, dividing the feature map into 8 paths reduces the channel dimension allocated to each path to *C*/8. This overly constrains the representation capability of the Mamba block, leaving it with insufficient channel capacity to learn robust and diverse feature representations. Therefore, we adopted the 4-path configuration as the optimal design, as it strikes the best balance between feature diversity and representation quality.

**Table 3:** Ablation study on the number of parallel paths in the MPM module. The best results are highlighted in bold.

Paths	Dice	IoU	Precision	SE
**DDT1 Dataset**				
2 Paths	76.88 ± 0.24	64.53 ± 0.17	80.92 ± 0.28	80.15 ± 0.13
**4 Paths (Ours)**	**77.45 ± 0.22**	**65.11 ± 0.25**	**83.11 ± 0.16**	**80.92 ± 0.28**
8 Paths	77.12 ± 0.18	64.78 ± 0.26	82.83 ± 0.20	80.53 ± 0.24
**TG3K Dataset**				
2 Paths	97.93 ± 0.26	95.59 ± 0.21	96.52 ± 0.19	98.37 ± 0.29
**4 Paths (Ours)**	**98.02 ± 0.14**	**96.01 ± 0.23**	**97.25 ± 0.29**	**98.45 ± 0.17**
8 Paths	97.98 ± 0.12	95.88 ± 0.28	97.01 ± 0.15	98.41 ± 0.22
**TN3K Dataset**				
2 Paths	78.55 ± 0.15	68.49 ± 0.27	80.83 ± 0.23	83.21 ± 0.18
**4 Paths (Ours)**	**79.28 ± 0.24**	**69.34 ± 0.19**	**82.85 ± 0.27**	**83.01 ± 0.21**
8 Paths	79.01 ± 0.29	69.03 ± 0.16	81.92 ± 0.25	83.11 ± 0.13

Table notes: 1) Abbreviations: SE = Sensitivity, IoU = Intersection over Union, MPM = Multi-Path Parallel Mamba; 2) All metrics are presented as percentages (%).

## Discussion

**Fig 5:**
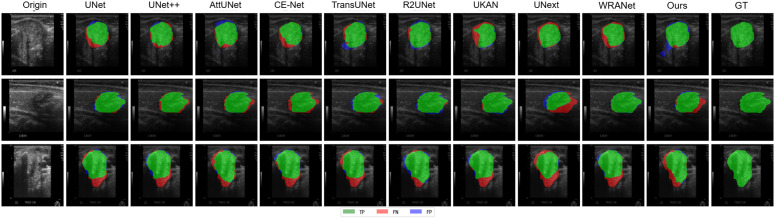
DDTI results. Visual comparison of segmentation results of our proposed PM-DUNet and other SOTA methods on the DDT1 dataset. From left to right: original image, segmentation results from different methods, and the Ground Truth.

**Fig 6:**
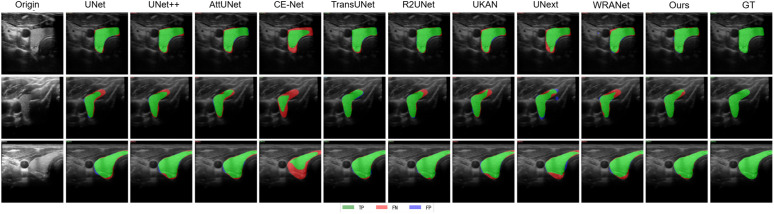
TG3K results. Visual comparison of segmentation results of our proposed PM-DUNet and other SOTA methods on the TG3K dataset. From left to right: original image, segmentation results from different methods, and the Ground Truth.

**Fig 7:**
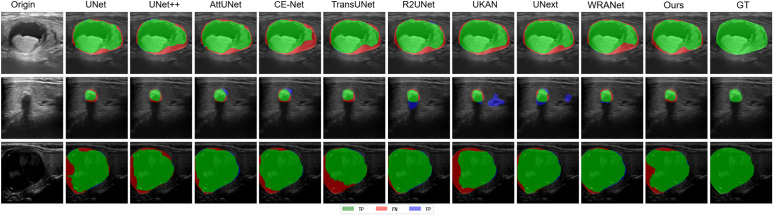
TN3K results. Visual comparison of segmentation results of our proposed PM-DUNet and other SOTA methods on the TN3K dataset. From left to right: original image, segmentation results from different methods, and the Ground Truth.

As shown in Table 1, our proposed PM-DUNet outperforms comparative methods on the core performance metrics of Dice and IoU across the three public thyroid datasets (DDT1, TG3K, and TN3K), which demonstrates the effectiveness of our model design. This superior performance can be attributed to two main factors: the robustness of our unique dual U-Net encoder-decoder architecture and the efficiency of our meticulously designed MPM, SED, and SAU modules. Quantitatively, compared to the baseline U-Net, PM-DUNet achieves Dice and IoU scores of 77.45% and 65.11% on the DDT1 dataset, marking significant improvements of 2.6% and 2.83%, respectively. On the larger and higher-quality TG3K dataset, our model also performs the best, reaching a Dice score of 98.02%. More notably, on the most challenging TN3K dataset, where nodules are smaller and boundaries are more ambiguous, our model still attains the highest Dice score of 79.28% and an IoU score of 69.34%, comprehensively surpassing other models on core indicators. Although WRANet achieves a slightly higher SE score on this dataset, our model delivers the best results on the comprehensive core metrics of Dice and IoU, which are more indicative of overall segmentation quality, thus proving its superior balanced performance. The architectural design of PM-DUNet plays a crucial role in its performance. First, the **dual U-Net cascade structure**, through a two-stage process of “coarse-to-fine” segmentation, enables the network to effectively correct potential biases from the initial stage, leading to more precise localization of nodule boundaries. Second, the **MPM module** used in the skip connections, with its powerful long-range dependency modeling capability, effectively captures the global context of the nodules and their surrounding tissues, addressing the limitations of traditional convolutions in understanding large-scale spatial relationships. Finally, the integrated **SED and SAU module** in the downsampling and upsampling paths allows the model to adaptively focus on the most informative feature channels and spatial regions, enhancing the representation of key features while suppressing irrelevant background noise. The synergy of these modules endows our model with strong accuracy and robustness when processing thyroid nodules of varying sizes and morphologies. The visual comparison of the experimental results is shown in Fig 5, Fig 6, and Fig 7.

## Conclusion

In this paper, we introduced a novel medical image segmentation framework, the PM-DUNet, designed to address the challenges inherent in thyroid ultrasound image segmentation, such as ambiguous nodule boundaries, variable sizes, and low contrast. The core of PM-DUNet is a cascaded dual U-Net encoder-decoder architecture, which effectively enhances segmentation accuracy and robustness through a two-stage “coarse-to-fine” strategy. The effectiveness of our work is primarily attributed to three meticulously designed modules. First, the MPM module is innovatively integrated into the skip connections. It leverages the strengths of SSMs to efficiently capture global context and long-range dependencies with linear computational complexity, which is crucial for accurately delineating ambiguous lesion borders. Second, our designed SED and SAU modules, by incorporating channel and spatial attention mechanisms, enable the network to adaptively focus on critical information during feature scale transformation, thereby enhancing the representation of effective features while suppressing irrelevant noise. Comprehensive quantitative and qualitative analyses on three public thyroid datasets (DDT1, TG3K, and TN3K) demonstrate that PM-DUNet outperforms existing state-of-the-art segmentation methods on most core performance metrics. Despite these advancements, our model has certain limitations that warrant further investigation. On one hand, although the MPM module itself is efficient, the overall dual U-Net architecture results in a relatively high parameter count, which may constrain its deployment on resource-limited devices. On the other hand, the model’s generalization capability has not yet been validated on other medical imaging modalities, such as CT or MRI. In future work, we plan to pursue improvements in two directions. First, we will explore model lightweighting techniques, such as knowledge distillation or network pruning, to reduce the model size while maintaining high performance, making it more accessible for clinical applications. Second, we aim to extend the PM-DUNet framework to a broader range of medical image segmentation tasks to further validate its generalization ability and applicability.

## Supporting information

S1 Fig PM-DUNet. The overall architecture of our proposed PM-DUNet.
(TIFF)

S2 Fig MPM module. Detailed structure of our proposed MPM module.
(TIFF)

S3 Fig SED module. The architecture of our proposed SED module.
(TIFF)

S4 Fig SAU module. The architecture of our proposed SAU module.
(TIFF)

S5 Fig DDTI results. Visual comparison of segmentation results of our proposed PM-DUNet and other SOTA methods on the DDT1 dataset.
(TIFF)

S6 Fig TG3K results. Visual comparison of segmentation results of our proposed PM-DUNet and other SOTA methods on the TG3K dataset.
(TIFF)

S7 Fig TN3K results. Visual comparison of segmentation results of our proposed PM-DUNet and other SOTA methods on the TN3K dataset.
(TIFF)

S1 Table Quantitative comparison of different models on DDT1, TG3K, and TN3K datasets. The best results are highlighted in bold.
(PNG)

S2 Table Component ablation study of our proposed PM-DUNet. The best results are highlighted in bold.
(PNG)

S3 Table Ablation study on the number of parallel paths in the MPM module. The best results are highlighted in bold.
(PNG)
