# Supplementary figures and images for: PM-DUnet: Fusing long-range dependencies and attention in a dual-U architecture for thyroid nodule segmentation
Source: PLoS One. 2026 Jul 30;21(7):e0353684. doi: 10.1371/journal.pone.0353684 (PMC13423043; doi:10.1371/journal.pone.0353684)

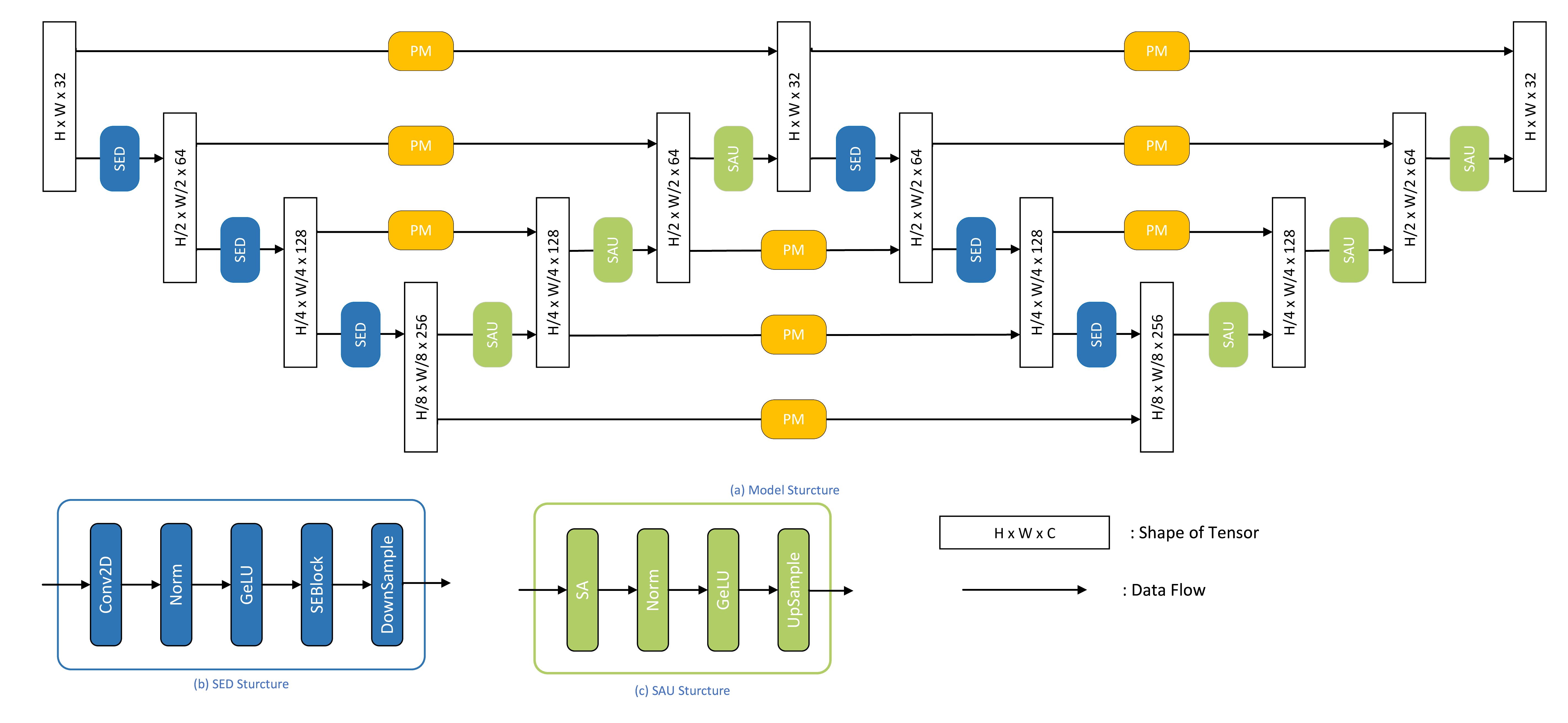

Supplement: S1 Fig — The overall architecture of our proposed PM-DUNet. (TIFF) [file pone.0353684.s001.tiff]

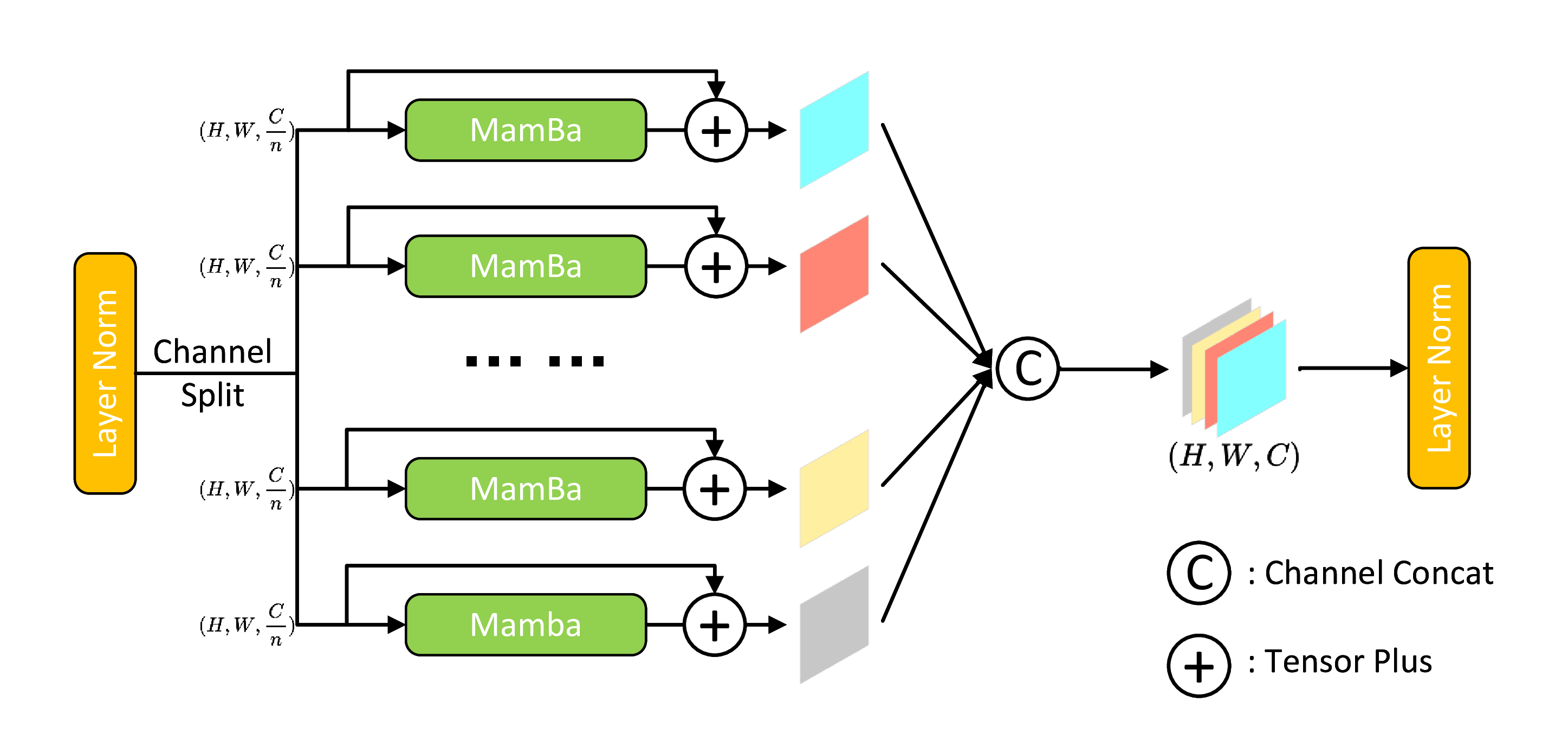

Supplement: S2 Fig — Detailed structure of our proposed MPM module. (TIFF) [file pone.0353684.s002.tiff]

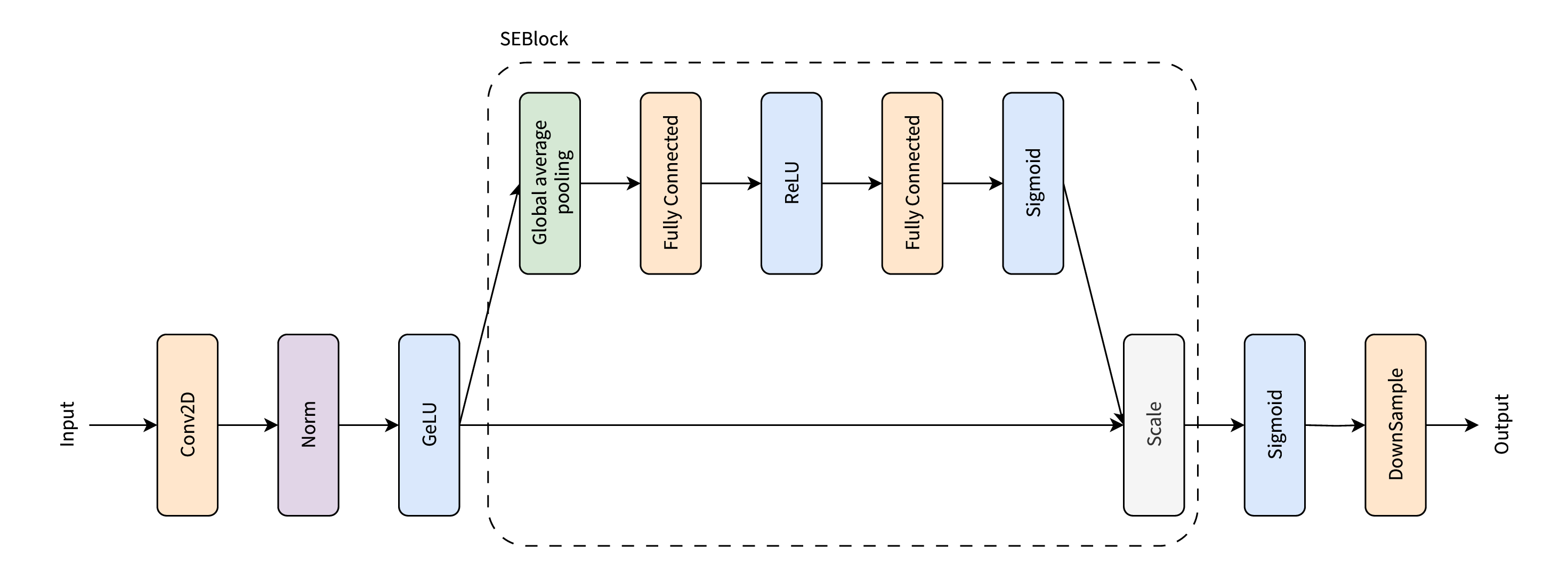

Supplement: S3 Fig — The architecture of our proposed SED module. (TIFF) [file pone.0353684.s003.tiff]

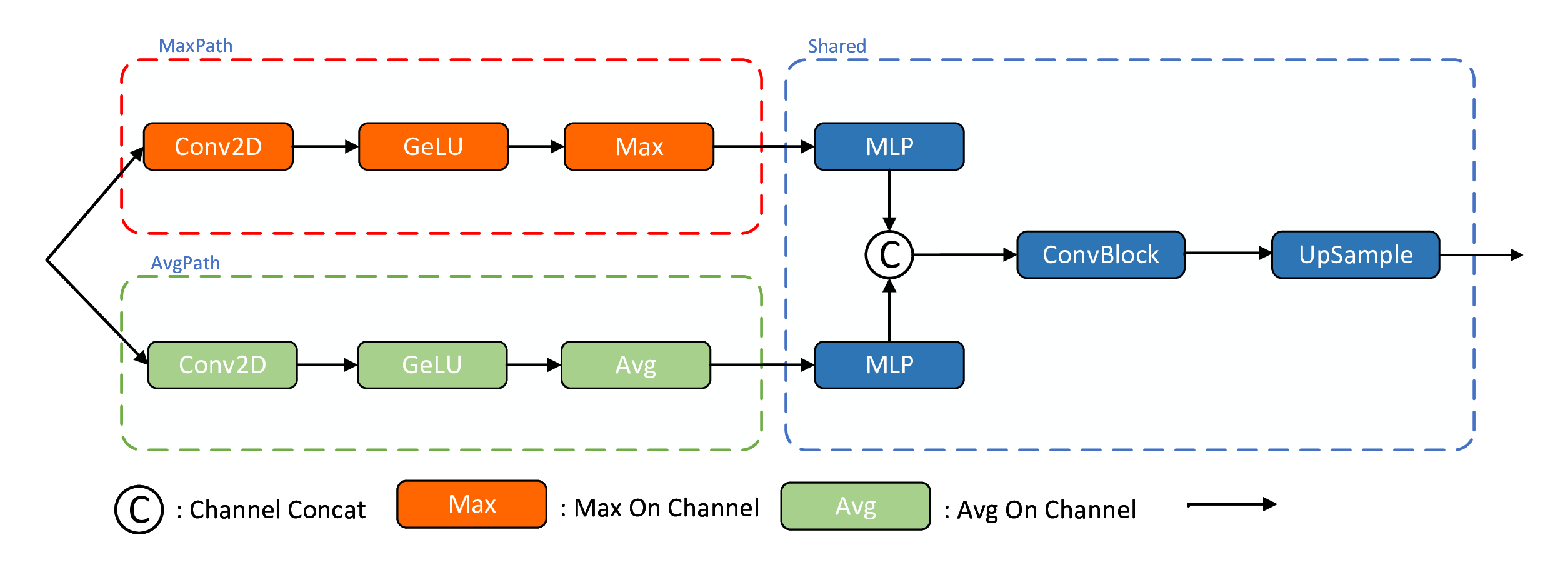

Supplement: S4 Fig — The architecture of our proposed SAU module. (TIFF) [file pone.0353684.s004.tiff]

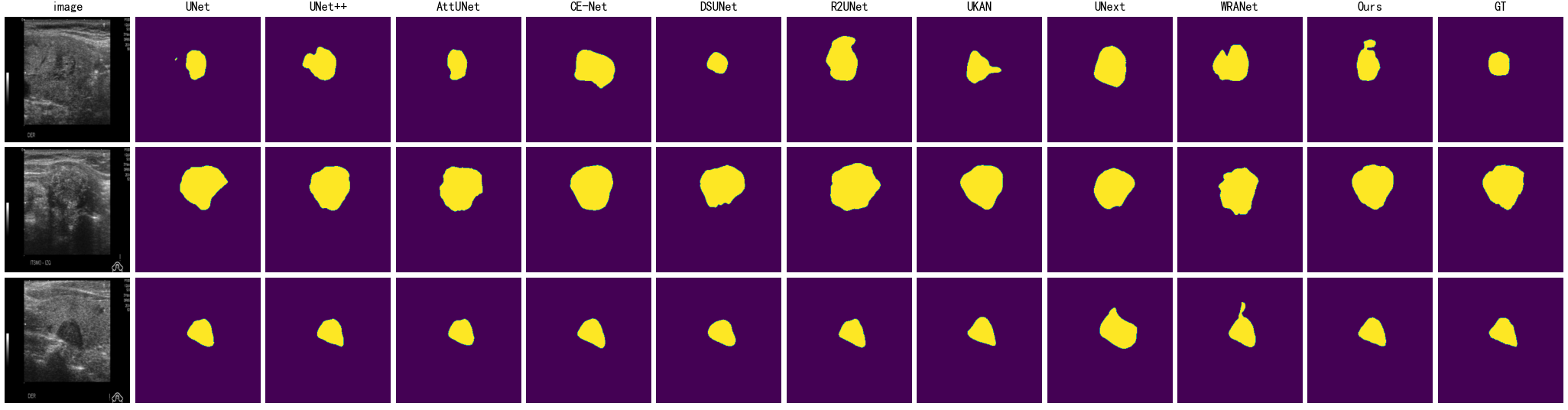

Supplement: S5 Fig — Visual comparison of segmentation results of our proposed PM-DUNet and other SOTA methods on the DDT1 dataset. (TIFF) [file pone.0353684.s005.tiff]

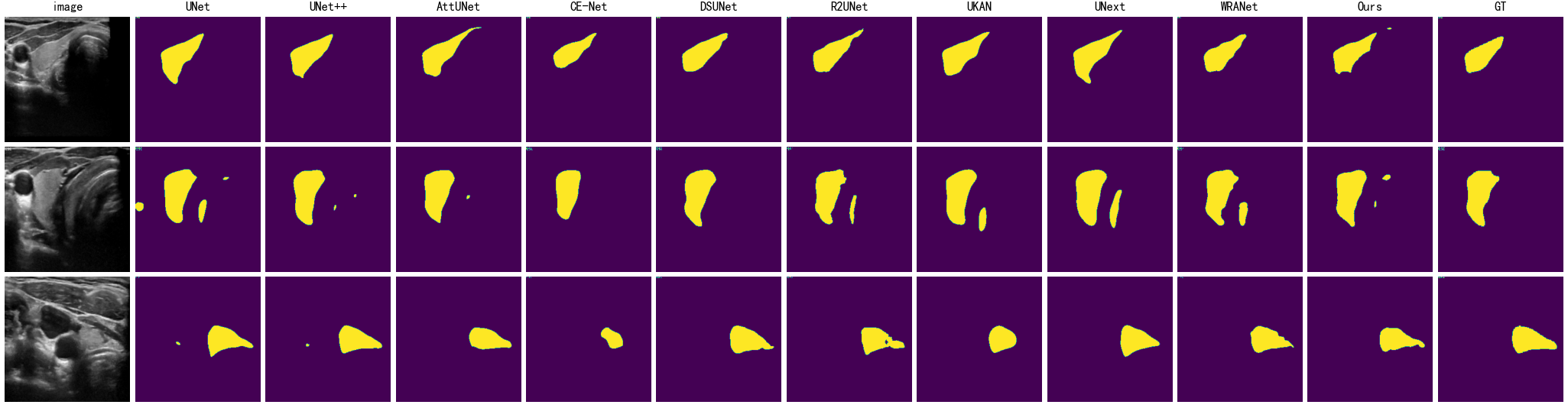

Supplement: S6 Fig — Visual comparison of segmentation results of our proposed PM-DUNet and other SOTA methods on the TG3K dataset. (TIFF) [file pone.0353684.s006.tiff]

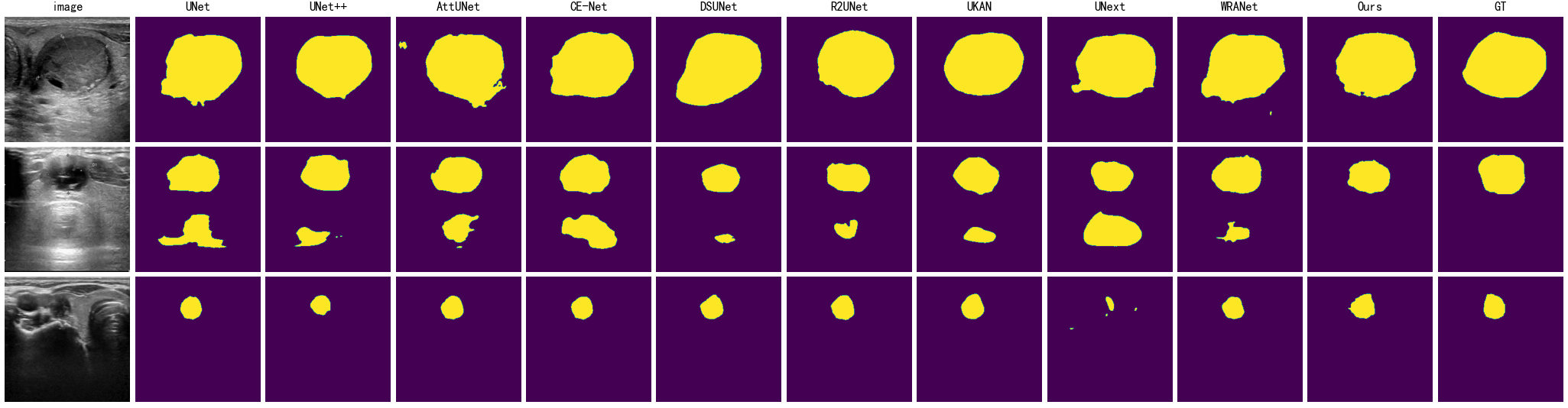

Supplement: S7 Fig — Visual comparison of segmentation results of our proposed PM-DUNet and other SOTA methods on the TN3K dataset. (TIFF) [file pone.0353684.s007.tiff]

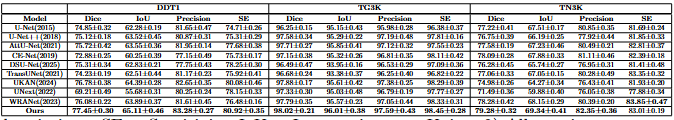

Supplement: S1 Table — The best results are highlighted in bold. (PNG) [file pone.0353684.s008.png]

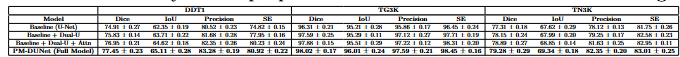

Supplement: S2 Table — The best results are highlighted in bold. (PNG) [file pone.0353684.s009.png]

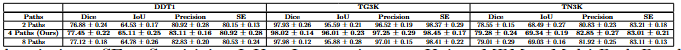

Supplement: S3 Table — The best results are highlighted in bold. (PNG) [file pone.0353684.s010.png]
