# Supplementary material for: PM-DUnet: Fusing long-range dependencies and attention in a dual-U architecture for thyroid nodule segmentation
Source: PLoS One. 2026 Jul 30;21(7):e0353684. doi: 10.1371/journal.pone.0353684 (PMC13423043; doi:10.1371/journal.pone.0353684)
Supplement: Attachment — Submitted filename: response to reviewer.docx [file pone.0353684.s012.docx]

**Response to Reviewers**

**Manuscript ID: [PONE-D-25-53692] - [EMID:fc80d242f4867e98]**

**Title: PM-DUnet:Fusing Long-Range Dependencies and Attention in a Dual-U Architecture for Thyroid Nodule Segmentation**

**Authors: Shaoqiang Wang et al.**

We would like to express our sincere gratitude for the time and effort you have dedicated to reviewing our manuscript. We have found the comments to be highly constructive and insightful, which have significantly helped us improve the quality, clarity, and rigor of our work.

**Response to Reviewer #1:**

We would like to thank the reviewer for pointing out this issue. Regarding the lack of training parameters and architectural information in the Methods section, we have supplemented the relevant details and organized them into a configuration table to ensure the reproducibility of the study:

**1. implementation details:**

Training Parameters:

Batch size (16 for the training set, 8 for the test set), optimizer (Adam), initial learning rate (0.0001), weight decay (1e-5), number of training epochs (200), loss function (BCEDiceLoss, combining binary cross-entropy and Dice loss).

Hardware Environment: NVIDIA RTX4090; CUDA-enabled device was used for training.

Software Environment: Python 3.13, PyTorch 2.7.1, CUDA 12.8

**2. Code availability:**

We appreciate the reviewer's reminder regarding code accessibility. To comply with PLOS ONE's reproducibility standards, we have uploaded the complete source code to a public repository (GitHub link: https://github.com/Andrevict/MPDUNet), which includes:

Training script (train.py) and model definition (Model.py);

Dataset loading code (dataset.py);

Implementation of evaluation metrics (evaluate.py) and loss function (loss.py);

Inference script (inference.py) and environment dependency file (requirements.txt).

All codes are accompanied by detailed comments to ensure researchers can reproduce the experimental results.

**3. Segmentation Result Visualization**

In response to the suggestion for revising the qualitative analysis figures, we have optimized Figures 5–7 as follows:

Added pixel-wise overlay maps to mark error regions with colors: green for True Positives (TP), red for False Negatives (FN), and blue for False Positives (FP);

Highlighted segmentation errors at nodule boundaries to enhance the distinguishability of different types of errors and improve the intuitiveness of result interpretation.

The revised figures will more clearly demonstrate the model's performance in boundary regions.

We would like to express our sincere gratitude again for the reviewer's valuable comments, which have helped improve the completeness and interpretability of our study. We have revised the manuscript as requested, and the relevant supplementary content has been marked at the corresponding positions.

**Response to Reviewer #2:**

We would like to thank the reviewer for pointing out this issue.

**1 Explanation of Data Segmentation Granularity**

Regarding the issue that the data segmentation failed to clearly specify whether it is "patient - level" or "image - level", we have supplemented detailed explanations in the methodology section. This study adopts patient - level split to divide all datasets into the training set and the test set. Specifically, the division is first performed based on the patient dimension to ensure that all images belonging to the same patient are assigned exclusively to either the training set or the test set, with no cross - set distribution. This segmentation method fundamentally avoids the data leakage problem that may be caused by image - level segmentation, thus ensuring the objectivity and reliability of the model performance evaluation. The relevant supplementary descriptions have been added to the "Dataset Partitioning" subsection.

**2 Supplementary Description of the Variability of Performance Metrics**

Addressing the problem that Tables 1 - 3 only provided single - point estimates and lacked variability indicators, we have re-analyzed and supplemented the experimental results. The experiment was repeated 20 times by varying the random seeds, and the mean ± standard deviation of each performance metric were calculated and supplemented in the tables. For example, the revised Tables 1-3 will present comprehensive information of the model on each dataset, which can reflect the stability of the model performance more comprehensively. The revised Tables 1-3 have replaced the corresponding original tables.

**3 Supplementary Citation of the TN3K Dataset Source**

Regarding the issue that the source of the TN3K dataset was not cited, we have supplemented its official source and the corresponding referenced literatures

**Response to Reviewer #3:**

We would like to thank the reviewer for their constructive comments. The issue you pointed out—that "a single random training/test split may affect the reliability of results"—is highly pertinent. To enhance the credibility and stability of the research conclusions, we have optimized the experimental design as suggested, with the specific modifications detailed below:

**1	Supplementary Design of Multi-Round Validation Experiments**

Addressing the limitation of the original experiment being based solely on a "single random training/test split," we have added repeated experiments with multiple random seeds:

independent random seeds were set to conduct 20 rounds of training and validation.

All experiments maintained the original training parameters unchanged to ensure the fairness of the validation results.

**2	Updated Presentation of Performance Metrics**

We have re-analyzed all experimental results, replacing the "single-point estimates" in the original tables with "mean ± standard deviation" to comprehensively reflect the stability of the model performance.

The performance metrics of the comparative models have also been supplemented with mean and standard deviation values to ensure the rigor of horizontal comparisons.

The revised tables have replaced the corresponding content in the original manuscript. The newly added statistical results demonstrate that our proposed PM-DUnet exhibits excellent robustness across different data splits, further verifying the reliability of its segmentation performance.

**Response to Reviewer #4:**

We sincerely appreciate the reviewer's careful review and valuable suggestions. The issues you raised regarding experimental design, reproducibility, baseline model comparisons, and reference citations are of great significance for enhancing the rigor and persuasiveness of this study. We have verified each point and made targeted revisions, with detailed explanations as follows:

**1	Optimization of Data Splitting and Validation Strategy**

Addressing the limitation of the original experiment that only adopted a "single 8:2 random split" without an independent validation set, we have comprehensively optimized the data splitting and validation strategy. The specific revisions are as follows:

Data Splitting: The dataset is divided into a training set and a test set at an 8:2 ratio, using stratified sampling based on the patient dimension to avoid data leakage.

Hyperparameter Tuning: All hyperparameters are determined based on the performance on the validation set, and an early stopping strategy is employed to prevent overfitting, ensuring the model's generalization ability.

Multi-Round Repeated Validation: 20 independent random seeds are set, and validation is performed under each seed. All performance metrics are presented as "mean ± standard deviation" to fully reflect the model's stability.

**2	Supplementary Training Details and Code Availability**

To address the issues of opaque training details and insufficient reproducibility, we have improved the work from two aspects: "information supplementation" and "code availability":

Supplementary Core Training Details: All key training parameters are summarized in Table 1, and the specific definition of "consistent experimental settings" is clarified.

Improved Code Availability: The complete source code has been made publicly available on a GitHub repository.

The repository includes a full dependency file, enabling direct verification of the experiment's authenticity.

**3	Addition of Transformer-Based Baseline Model Comparisons**

Thank you for pointing out the limitation of the original baseline model comparisons. To verify the efficiency advantage of PM-DUnet in handling long-range dependencies, we have added one mainstream Transformer-based medical segmentation model as a new baseline. The supplementary experimental results are as follows:

Newly Added Baseline Models: TransUNet which are widely used in the field, are selected for fair comparison, strictly following the original experimental settings.

Conclusion Support: The supplementary results show that PM-DUnet outperforms TransUNet in IoU, directly verifying the core argument that "the proposed method handles long-range dependencies more efficiently." Through the MPM module, PM-DUnet achieves superior segmentation performance while avoiding the complex self-attention computation of Transformers.

**4	Correction of Reference Citations**

We would like to thank the reviewer for accurately identifying this academic standard issue. We sincerely apologize for the oversight in citing Reference [9] (Hässler et al., 2022), which is misaligned with the context of "ambiguous boundaries of thyroid nodules in ultrasound imaging." We have thoroughly revised this issue, and the detailed explanations are as follows:

We have removed the citation of Reference [9] (Hässler et al., 2022) from the original manuscript. This study focuses on model calibration and out-of-distribution detection in segmentation tasks, with no direct relevance to ultrasound imaging or nodule boundary ambiguity. As such, it fails to support the claim that "ambiguous boundaries are a core challenge in thyroid nodule segmentation," making it an inappropriate citation.
