# Supplementary material for: PM-DUnet: Fusing long-range dependencies and attention in a dual-U architecture for thyroid nodule segmentation
Source: PLoS One. 2026 Jul 30;21(7):e0353684. doi: 10.1371/journal.pone.0353684 (PMC13423043; doi:10.1371/journal.pone.0353684)
Supplement: Attachment — Submitted filename: Response.docx [file pone.0353684.s013.docx]

Dear Editor and Reviewers,

We would like to express our sincere gratitude to the editor for giving us the opportunity to revise our manuscript. We are deeply thankful to all reviewers for their constructive, detailed, and valuable comments, which have been of great importance in improving the quality of our manuscript.

**Response to Reviewer #4**

**Comment 1**: Contradiction about dataset split (random split vs. patient-level split)

**Response:** We apologize for the contradictory description in the original manuscript. To avoid data leakage and ensure the validity of experimental results, we strictly adopted patient-level splitting for all datasets in our experiments. We have revised the corresponding sentence in the manuscript. All inconsistent descriptions have been unified and corrected to ensure the experimental procedure is clearly and accurately reported.

**Comment 2**: Unclear validation set / independent test set / early stopping

**Response:** We sincerely apologize for the unclear description. We clarify as follows:

The dataset was partitioned into a training set (80%) and a test set (20%) at the patient level. Early stopping was adopted based on the loss on the validation set, with a patience of 15 epochs.

**Comment 3**: Contradiction on image resizing (256×256 vs. 224×224)

**Response:** We apologize for this error. All images in our experiments were uniformly resized to 224 × 224 pixels throughout the preprocessing. We have deleted the incorrect description of “256 × 256” and ensured that all parts of the manuscript are consistent with 224 × 224 pixels.

**Comment 4**: Missing data augmentation

**Response**: We have added the missing experimental details in the Materials and methods section: We don’t applied any data augmentation during training.

**Comment 5**: Missing description of repeated experiments (20 times with random seeds)

**Response**: We have added the following description in the manuscript:

Each experiment was repeated 20 times with different random seeds, and the performance metrics are reported as the mean ± standard deviation.

This ensures consistency between the text and the tables.

**Comment 6**: Acknowledge that WRANet has higher specificity (SE) on TN3K

**Response**: We agree with the reviewer. We have revised the Discussion section to acknowledge that. This makes the conclusion more objective and rigorous.

Thank you again for your valuable comments.
