# Supplementary material for: PM-DUnet: Fusing long-range dependencies and attention in a dual-U architecture for thyroid nodule segmentation
Source: PLoS One. 2026 Jul 30;21(7):e0353684. doi: 10.1371/journal.pone.0353684 (PMC13423043; doi:10.1371/journal.pone.0353684)
Supplement: Attachment — Submitted filename: Reponse.docx [file pone.0353684.s014.docx]

**Response to Reviewer #4**

**Point 1:** *The training/validation/test protocol is contradictory and not reproducible...*

**Response:** We sincerely apologize for the confusion caused by the contradictory statements regarding our training protocol. We confirm that the dataset was partitioned at the patient level into an 80/20 split for training and testing, respectively. Early stopping was indeed implemented based on the validation loss with a patience of 15 epochs. We have corrected the Results section in the manuscript to accurately reflect this protocol and have removed any ambiguous phrasing.

**Point 2:** *The method section states: “Each subsequence xk is then processed independently by a weight-shared Mamba block.” Later... explains the performance drop as follows: “attributed to excessive parameter fragmentation”...*

**Response:** We appreciate your careful reading and constructive feedback. because the parallel paths share parameters, the overall parameter count per block does not decrease. Rather, dividing the feature map into 8 paths reduces the channel dimension processed by the shared block to C/8. This drastically limits the block's representation capacity, leaving it with too few channels to learn robust and meaningful feature relationships. We have revised the Ablation Study section to accurately explain this dynamic, and we have included the exact parameter counts for the 2-, 4-, and 8-path variants to provide complete transparency.

**Point 3:** *In Figure 4 and its caption state: “The module processes features through MaxPath, AvgPath, and a shared MLP layer.” However, the mathematical description defines the attention map as... a convolution-based spatial attention mechanism...*

**Response:** Thank you for pointing out this inconsistency. The mathematical description outlining a 7x7 convolutional layer is correct. The mention of a "shared MLP" in the figure caption was an oversight carried over from a previous draft of the architecture. We have updated Figure 4's caption to correctly state that the module utilizes a convolutional layer.

**Point 4:** *The manuscript states: “TG3K Dataset: ... comprising 3478 ultrasound images from 3478 patients” and “TN3K Dataset: ... 3108 thyroid ultrasound images from 3108 patients.” Yet published descriptions...*

**Response:** We apologize for the inaccuracies regarding the dataset statistics. We have double-checked the numbers with the original dataset publications and corrected the manuscript. The TG3K dataset description now correctly states it contains 3,583 images extracted from video sequences, and the TN3K description has been corrected to 3,493 images from 2,421 patients.

**Point 5:** *The main superiority claim is overstated relative to the manuscript’s own evidence...*

**Response:** We agree that our previous wording was slightly overstated, particularly given that WRANet achieves a higher Sensitivity score on the TN3K dataset. We have revised the Abstract and Conclusion to moderate these claims. The text now states that our method achieves highly competitive performance and outperforms state-of-the-art methods on *most* key metrics (such as Dice and IoU), ensuring our claims align accurately with the experimental evidence.
