# Supplementary material for: PM-DUnet: Fusing long-range dependencies and attention in a dual-U architecture for thyroid nodule segmentation
Source: PLoS One. 2026 Jul 30;21(7):e0353684. doi: 10.1371/journal.pone.0353684 (PMC13423043; doi:10.1371/journal.pone.0353684)
Supplement: Peer review history

# Peer review: Decision Letter 0

**DOI:** 10.1371/journal.pone.0353684.r001

**Authors:** Amirreza Khalaji

4 Nov 2025

PONE-D-25-53692

PM-DUnet:Fusing Long-Range Dependencies and Attention in a Dual-U Architecture for Thyroid Nodule Segmentation

PLOS ONE

Dear Dr. Cheng,

Thank you for submitting your manuscript to PLOS ONE. After careful consideration, we feel that it has merit but does not fully meet PLOS ONE’s publication criteria as it currently stands. Therefore, we invite you to submit a revised version of the manuscript that addresses the points raised during the review process.

Please submit your revised manuscript by Dec 19 2025 11:59PM. If you will need more time than this to complete your revisions, please reply to this message or contact the journal office at plosone@plos.org. When you're ready to submit your revision, log on to https://www.editorialmanager.com/pone/ and select the 'Submissions Needing Revision' folder to locate your manuscript file.

Please include the following items when submitting your revised manuscript:

- A rebuttal letter that responds to each point raised by the academic editor and reviewer(s). You should upload this letter as a separate file labeled 'Response to Reviewers'.
- A marked-up copy of your manuscript that highlights changes made to the original version. You should upload this as a separate file labeled 'Revised Manuscript with Track Changes'.
- An unmarked version of your revised paper without tracked changes. You should upload this as a separate file labeled 'Manuscript'.

-->If you would like to make changes to your financial disclosure, please include your updated statement in your cover letter. Guidelines for resubmitting your figure files are available below the reviewer comments at the end of this letter.

If applicable, we recommend that you deposit your laboratory protocols in protocols.io to enhance the reproducibility of your results. Protocols.io assigns your protocol its own identifier (DOI) so that it can be cited independently in the future. For instructions see: https://journals.plos.org/plosone/s/submission-guidelines#loc-laboratory-protocols. Additionally, PLOS ONE offers an option for publishing peer-reviewed Lab Protocol articles, which describe protocols hosted on protocols.io. Read more information on sharing protocols at https://plos.org/protocols?utm_medium=editorial-email&utm_source=authorletters&utm_campaign=protocols.

We look forward to receiving your revised manuscript.

Kind regards,

Amirreza Khalaji

Academic Editor

PLOS ONE

Journal Requirements:

When submitting your revision, we need you to address these additional requirements.

1. Please ensure that your manuscript meets PLOS ONE's style requirements, including those for file naming. The PLOS ONE style templates can be found at

https://journals.plos.org/plosone/s/file?id=wjVg/PLOSOne_formatting_sample_main_body.pdf and

https://journals.plos.org/plosone/s/file?id=ba62/PLOSOne_formatting_sample_title_authors_affiliations.pdf

2. Please note that PLOS One has specific guidelines on code sharing for submissions in which author-generated code underpins the findings in the manuscript. In these cases, we expect all author-generated code to be made available without restrictions upon publication of the work. Please review our guidelines at https://journals.plos.org/plosone/s/materials-and-software-sharing#loc-sharing-code and ensure that your code is shared in a way that follows best practice and facilitates reproducibility and reuse.

3. Thank you for stating the following in the Acknowledgments Section of your manuscript:

“Authors are funded by UKRI Grant EP/W020408/1 and Grant RS718 through 394 Doctoral Training Centre at Swansea University.”

We note that you have provided funding information that is not currently declared in your Funding Statement. However, funding information should not appear in the Acknowledgments section or other areas of your manuscript. We will only publish funding information present in the Funding Statement section of the online submission form.

Please remove any funding-related text from the manuscript and let us know how you would like to update your Funding Statement. Currently, your Funding Statement reads as follows:

“Authors are funded by UKRI Grant EP/W020408/1 and Grant RS718 through 394 Doctoral Training Centre at Swansea University”

Please include your amended statements within your cover letter; we will change the online submission form on your behalf.

4. Thank you for stating the following financial disclosure:

“Authors are funded by UKRI Grant EP/W020408/1 and Grant RS718 through 394 Doctoral Training Centre at Swansea University”

Please state what role the funders took in the study.  If the funders had no role, please state: "The funders had no role in study design, data collection and analysis, decision to publish, or preparation of the manuscript."

If this statement is not correct you must amend it as needed.

Please include this amended Role of Funder statement in your cover letter; we will change the online submission form on your behalf.

5. PLOS requires an ORCID iD for the corresponding author in Editorial Manager on papers submitted after December 6th, 2016. Please ensure that you have an ORCID iD and that it is validated in Editorial Manager. To do this, go to ‘Update my Information’ (in the upper left-hand corner of the main menu), and click on the Fetch/Validate link next to the ORCID field. This will take you to the ORCID site and allow you to create a new iD or authenticate a pre-existing iD in Editorial Manager.

6. Please ensure that you refer to Figure 1 - 3 in your text as, if accepted, production will need this reference to link the reader to the figure.

7. We note you have included a table to which you do not refer in the text of your manuscript. Please ensure that you refer to Table 2 and 3 in your text; if accepted, production will need this reference to link the reader to the Table.

8. Please upload a copy of Supporting Information Figure S1, S2, S3 and S4 and Table S1, S2 and S3 which you refer to in your text on page 13.

9. If the reviewer comments include a recommendation to cite specific previously published works, please review and evaluate these publications to determine whether they are relevant and should be cited. There is no requirement to cite these works unless the editor has indicated otherwise.

10. Please review your reference list to ensure that it is complete and correct. If you have cited papers that have been retracted, please include the rationale for doing so in the manuscript text, or remove these references and replace them with relevant current references. Any changes to the reference list should be mentioned in the rebuttal letter that accompanies your revised manuscript. If you need to cite a retracted article, indicate the article’s retracted status in the References list and also include a citation and full reference for the retraction notice.

[Note: HTML markup is below. Please do not edit.]

Reviewers' comments:

Reviewer's Responses to Questions

**Comments to the Author**

1. Is the manuscript technically sound, and do the data support the conclusions?

The manuscript must describe a technically sound piece of scientific research with data that supports the conclusions. Experiments must have been conducted rigorously, with appropriate controls, replication, and sample sizes. The conclusions must be drawn appropriately based on the data presented.

Reviewer #1: Yes

Reviewer #2: Yes

Reviewer #3: Yes

Reviewer #4: Partly

**********

2. Has the statistical analysis been performed appropriately and rigorously?

Reviewer #1: Yes

Reviewer #2: Yes

Reviewer #3: Yes

Reviewer #4: No

**********

3. Have the authors made all data underlying the findings in their manuscript fully available?

The PLOS Data policy requires authors to make all data underlying the findings described in their manuscript fully available without restriction, with rare exception (please refer to the Data Availability Statement in the manuscript PDF file). The data should be provided as part of the manuscript or its supporting information, or deposited to a public repository. For example, in addition to summary statistics, the data points behind means, medians and variance measures should be available. If there are restrictions on publicly sharing data—e.g. participant privacy or use of data from a third party—those must be specified.

Reviewer #1: Yes

Reviewer #2: Yes

Reviewer #3: Yes

Reviewer #4: No

**********

4. Is the manuscript presented in an intelligible fashion and written in standard English?

PLOS ONE does not copyedit accepted manuscripts, so the language in submitted articles must be clear, correct, and unambiguous. Any typographical or grammatical errors should be corrected at revision, so please note any specific errors here.

Reviewer #1: Yes

Reviewer #2: Yes

Reviewer #3: Yes

Reviewer #4: No

**********

5. Review Comments to the Author

Please use the space provided to explain your answers to the questions above. You may also include additional comments for the author, including concerns about dual publication, research ethics, or publication ethics. (Please upload your review as an attachment if it exceeds 20,000 characters)

Reviewer #1: Missing implementation details:

The Methods section lacks essential training and architectural information (e.g., batch size, optimizer, learning rate, number of epochs, loss function, hardware, and software). These are necessary for reproducibility and should be clearly summarized in a configuration table.

Code availability:

The paper provides dataset links but no mention of source code. The authors should include a public code repository (e.g., GitHub) with training and inference scripts to comply with PLOS ONE’s reproducibility standards.

Segmentation visualization:

The qualitative figures (Figs. 5–7) should be revised to include pixel-wise overlays showing True Positives (green), False Negatives (red), and False Positives (blue). This would make the results more interpretable and clearly reveal segmentation errors along nodule boundaries.

Reviewer #2: Thank you for inviting me for this review. This is an interesting study, but there are few concerns that should be addressed before any decision.

Comments

1. Although the authors mention splitting the data into training and testing sets, they fail to clarify whether the split was done at the patient or image level. Image-level splitting could cause the same patient’s images to appear in both sets, leading to data leakage and artificially inflated performance metrics.

2. Tables 1–3 present performance results only as single point estimates, without measures of variability such as 95% confidence intervals or standard deviations.

3. In the Materials section, the TN3K dataset is described without providing a reference to its source, unlike DDTI and TG3K which cite sources [20] and [21].

Reviewer #3: The algorithm and architecture diagram are clear. However, I’m concerned about the reliability of your comparative results, since they’re based on a single random train/test split. Please rerun the experiments with multiple random seeds (or use k-fold cross-validation) and report aggregated metrics—for example, mean ± std—to make the comparison more trustworthy.

Reviewer #4: Thank you for your hard effort on developing and running this mode. Apart from some minor linguistic issues which should be considered, I have more profound concerns about the method applied. Some of which can be modified and some are not employable; since the work is done. My first concern is “For all the datasets mentioned above, we followed the standard practice in the literature by randomly splitting them into training and testing sets at an 8:2 ratio.”[1] This indicates the authors only performed a single random train-test split with no dedicated validation set or cross-validation. The lack of a validation set raises concerns about model selection and hyperparameter tuning. it’s unclear how they avoided overfitting or chose the final model parameters. Without multiple runs or a validation set, the reported performance on the test set may not be reliable or reproducible.

Missing Training Details and Reproducibility Concerns: The manuscript omits important training details, making it difficult to reproduce the results. For instance, there is no information about the optimizer type, learning rate, number of training epochs, batch size, or any regularization techniques used. The authors state that “All experiments were conducted under identical settings to ensure fair comparisons.” but they never define these settings anywhere in the paper. Without such information or a released code, one cannot confirm the training regimen or replicate the experiments. This lack of transparency in experimental setup and hyperparameters is a significant methodological flaw, as it hinders verification of the findings.

No Transformer-Based Baseline for Comparison: In the introduction, the authors emphasize the drawbacks of Transformers (complexity, data requirements) and motivate the use of state-space models. However, they do not include any transformer-based segmentation model in their experimental comparison. Established Transformer-based medical segmentation models (e.g. TransUNet or Swin-Unet) are notably absent from Table 1. Including at least one such method would have strengthened the evaluation – for example, to show that PM-DUNet achieves similar accuracy with fewer parameters or less compute. The omission means the claim that the proposed method handles long-range dependencies “more efficiently” than Transformers isn’t directly validated. It leaves a gap in the experimental evidence, since all the compared baselines are CNN-based or minor CNN/MLP variants, except DSU-Net which has a Transformer component. This limits the scope of the conclusions regarding the advantages of the MPM module over full self-attention mechanisms.

• Potentially Misused Reference: “…ambiguous boundaries, such as thyroid nodules in ultrasound imaging [9]…”. Reference [9] (Hässler et al., 2022) deals with model calibration and out-of-distribution detection in segmentation, which is not directly about ultrasound or ambiguous nodule boundaries. Citing it in the context of “ambiguous boundaries in thyroid US” appears misaligned. This reference does not provide evidence for the specific claim being made, so it may be inappropriate or at least not the best choice. The authors should ensure that references support the statements; if none exists for that claim, it might be better to remove or replace [9] with a more relevant citation (or simply state the challenge without citation, as it’s a known issue in ultrasound imaging).

**********

6. PLOS authors have the option to publish the peer review history of their article (what does this mean?). If published, this will include your full peer review and any attached files.

If you choose “no”, your identity will remain anonymous but your review may still be made public.

**Do you want your identity to be public for this peer review?**  For information about this choice, including consent withdrawal, please see our Privacy Policy.

Reviewer #1: No

Reviewer #2: No

Reviewer #3: No

Reviewer #4: No

**********

[NOTE: If reviewer comments were submitted as an attachment file, they will be attached to this email and accessible via the submission site. Please log into your account, locate the manuscript record, and check for the action link "View Attachments". If this link does not appear, there are no attachment files.]

To ensure your figures meet our technical requirements, please review our figure guidelines: https://journals.plos.org/plosone/s/figures

You may also use PLOS’s free figure tool, NAAS, to help you prepare publication quality figures: https://journals.plos.org/plosone/s/figures#loc-tools-for-figure-preparation.

NAAS will assess whether your figures meet our technical requirements by comparing each figure against our figure specifications.

# Author comment: Author response to Decision Letter 1

**DOI:** 10.1371/journal.pone.0353684.r002

23 Dec 2025

Response to Reviewers

Manuscript ID: [PONE-D-25-53692] - [EMID:fc80d242f4867e98]

Title: PM-DUnet:Fusing Long-Range Dependencies and Attention in a Dual-U Architecture for Thyroid Nodule Segmentation

Authors: Shaoqiang Wang et al.

We would like to express our sincere gratitude for the time and effort you have dedicated to reviewing our manuscript. We have found the comments to be highly constructive and insightful, which have significantly helped us improve the quality, clarity, and rigor of our work.

Response to Reviewer #1:

We would like to thank the reviewer for pointing out this issue. Regarding the lack of training parameters and architectural information in the Methods section, we have supplemented the relevant details and organized them into a configuration table to ensure the reproducibility of the study:

1. implementation details:

Training Parameters:

Batch size (16 for the training set, 8 for the test set), optimizer (Adam), initial learning rate (0.0001), weight decay (1e-5), number of training epochs (200), loss function (BCEDiceLoss, combining binary cross-entropy and Dice loss).

Hardware Environment: NVIDIA RTX4090; CUDA-enabled device was used for training.

Software Environment: Python 3.13, PyTorch 2.7.1, CUDA 12.8

2. Code availability:

We appreciate the reviewer's reminder regarding code accessibility. To comply with PLOS ONE's reproducibility standards, we have uploaded the complete source code to a public repository (GitHub link: https://github.com/Andrevict/MPDUNet), which includes:

Training script (train.py) and model definition (Model.py);

Dataset loading code (dataset.py);

Implementation of evaluation metrics (evaluate.py) and loss function (loss.py);

Inference script (inference.py) and environment dependency file (requirements.txt).

All codes are accompanied by detailed comments to ensure researchers can reproduce the experimental results.

3. Segmentation Result Visualization

In response to the suggestion for revising the qualitative analysis figures, we have optimized Figures 5–7 as follows:

Added pixel-wise overlay maps to mark error regions with colors: green for True Positives (TP), red for False Negatives (FN), and blue for False Positives (FP);

Highlighted segmentation errors at nodule boundaries to enhance the distinguishability of different types of errors and improve the intuitiveness of result interpretation.

The revised figures will more clearly demonstrate the model's performance in boundary regions.

We would like to express our sincere gratitude again for the reviewer's valuable comments, which have helped improve the completeness and interpretability of our study. We have revised the manuscript as requested, and the relevant supplementary content has been marked at the corresponding positions.

Response to Reviewer #2:

We would like to thank the reviewer for pointing out this issue.

1 Explanation of Data Segmentation Granularity

Regarding the issue that the data segmentation failed to clearly specify whether it is "patient - level" or "image - level", we have supplemented detailed explanations in the methodology section. This study adopts patient - level split to divide all datasets into the training set and the test set. Specifically, the division is first performed based on the patient dimension to ensure that all images belonging to the same patient are assigned exclusively to either the training set or the test set, with no cross - set distribution. This segmentation method fundamentally avoids the data leakage problem that may be caused by image - level segmentation, thus ensuring the objectivity and reliability of the model performance evaluation. The relevant supplementary descriptions have been added to the "Dataset Partitioning" subsection.

2 Supplementary Description of the Variability of Performance Metrics

Addressing the problem that Tables 1 - 3 only provided single - point estimates and lacked variability indicators, we have re-analyzed and supplemented the experimental results. The experiment was repeated 20 times by varying the random seeds, and the mean ± standard deviation of each performance metric were calculated and supplemented in the tables. For example, the revised Tables 1-3 will present comprehensive information of the model on each dataset, which can reflect the stability of the model performance more comprehensively. The revised Tables 1-3 have replaced the corresponding original tables.

3 Supplementary Citation of the TN3K Dataset Source

Regarding the issue that the source of the TN3K dataset was not cited, we have supplemented its official source and the corresponding referenced literatures

Response to Reviewer #3:

We would like to thank the reviewer for their constructive comments. The issue you pointed out—that "a single random training/test split may affect the reliability of results"—is highly pertinent. To enhance the credibility and stability of the research conclusions, we have optimized the experimental design as suggested, with the specific modifications detailed below:

1 Supplementary Design of Multi-Round Validation Experiments

Addressing the limitation of the original experiment being based solely on a "single random training/test split," we have added repeated experiments with multiple random seeds:

independent random seeds were set to conduct 20 rounds of training and validation.

All experiments maintained the original training parameters unchanged to ensure the fairness of the validation results.

2 Updated Presentation of Performance Metrics

We have re-analyzed all experimental results, replacing the "single-point estimates" in the original tables with "mean ± standard deviation" to comprehensively reflect the stability of the model performance.

The performance metrics of the comparative models have also been supplemented with mean and standard deviation values to ensure the rigor of horizontal comparisons.

The revised tables have replaced the corresponding content in the original manuscript. The newly added statistical results demonstrate that our proposed PM-DUnet exhibits excellent robustness across different data splits, further verifying the reliability of its segmentation performance.

Response to Reviewer #4:

We sincerely appreciate the reviewer's careful review and valuable suggestions. The issues you raised regarding experimental design, reproducibility, baseline model comparisons, and reference citations are of great significance for enhancing the rigor and persuasiveness of this study. We have verified each point and made targeted revisions, with detailed explanations as follows:

1 Optimization of Data Splitting and Validation Strategy

Addressing the limitation of the original experiment that only adopted a "single 8:2 random split" without an independent validation set, we have comprehensively optimized the data splitting and validation strategy. The specific revisions are as follows:

Data Splitting: The dataset is divided into a training set and a test set at an 8:2 ratio, using stratified sampling based on the patient dimension to avoid data leakage.

Hyperparameter Tuning: All hyperparameters are determined based on the performance on the validation set, and an early stopping strategy is employed to prevent overfitting, ensuring the model's generalization ability.

Multi-Round Repeated Validation: 20 independent random seeds are set, and validation is performed under each seed. All performance metrics are presented as "mean ± standard deviation" to fully reflect the model's stability.

2 Supplementary Training Details and Code Availability

To address the issues of opaque training details and insufficient reproducibility, we have improved the work from two aspects: "information supplementation" and "code availability":

Supplementary Core Training Details: All key training parameters are summarized in Table 1, and the specific definition of "consistent experimental settings" is clarified.

Improved Code Availability: The complete source code has been made publicly available on a GitHub repository.

The repository includes a full dependency file, enabling direct verification of the experiment's authenticity.

3 Addition of Transformer-Based Baseline Model Comparisons

Thank you for pointing out the limitation of the original baseline model comparisons. To verify the efficiency advantage of PM-DUnet in handling long-range dependencies, we have added one mainstream Transformer-based medical segmentation model as a new baseline. The supplementary experimental results are as follows:

Newly Added Baseline Models: TransUNet which are widely used in the field, are selected for fair comparison, strictly following the original experimental settings.

Conclusion Support: The supplementary results show that PM-DUnet outperforms TransUNet in IoU, directly verifying the core argument that "the proposed method handles long-range dependencies more efficiently." Through the MPM module, PM-DUnet achieves superior segmentation performance while avoiding the complex self-attention computation of Transformers.

4 Correction of Reference Citations

We would like to thank the reviewer for accurately identifying this academic standard issue. We sincerely apologize for the oversight in citing Reference [9] (Hässler et al., 2022), which is misaligned with the context of "ambiguous boundaries of thyroid nodules in ultrasound imaging." We have thoroughly revised this issue, and the detailed explanations are as follows:

We have removed the citation of Reference [9] (Hässler et al., 2022) from the original manuscript. This study focuses on model calibration and out-of-distribution detection in segmentation tasks, with no direct relevance to ultrasound imaging or nodule boundary ambiguity. As such, it fails to support the claim that "ambiguous boundaries are a core challenge in thyroid nodule segmentation," making it an inappropriate citation.

Attachment Submitted filename: response to reviewer.docx

# Peer review: Decision Letter 1

**DOI:** 10.1371/journal.pone.0353684.r003

**Authors:** Amirreza Khalaji

27 Jan 2026

PONE-D-25-53692R1

PM-DUnet:Fusing Long-Range Dependencies and Attention in a Dual-U Architecture for Thyroid Nodule Segmentation

PLOS One

Dear Dr. Cheng,

Thank you for submitting your manuscript to PLOS ONE. After careful consideration, we feel that it has merit but does not fully meet PLOS ONE’s publication criteria as it currently stands. Therefore, we invite you to submit a revised version of the manuscript that addresses the points raised during the review process.

Please submit your revised manuscript by Mar 13 2026 11:59PM. If you will need more time than this to complete your revisions, please reply to this message or contact the journal office at plosone@plos.org. When you're ready to submit your revision, log on to https://www.editorialmanager.com/pone/ and select the 'Submissions Needing Revision' folder to locate your manuscript file.

Please include the following items when submitting your revised manuscript:

- A letter that responds to each point raised by the academic editor and reviewer(s). You should upload this letter as a separate file labeled 'Response to Reviewers'.
- A marked-up copy of your manuscript that highlights changes made to the original version. You should upload this as a separate file labeled 'Revised Manuscript with Track Changes'.
- An unmarked version of your revised paper without tracked changes. You should upload this as a separate file labeled 'Manuscript'.

-->If you would like to make changes to your financial disclosure, please include your updated statement in your cover letter. Guidelines for resubmitting your figure files are available below the reviewer comments at the end of this letter.

If applicable, we recommend that you deposit your laboratory protocols in protocols.io to enhance the reproducibility of your results. Protocols.io assigns your protocol its own identifier (DOI) so that it can be cited independently in the future. For instructions see: https://journals.plos.org/plosone/s/submission-guidelines#loc-laboratory-protocols. Additionally, PLOS ONE offers an option for publishing peer-reviewed Lab Protocol articles, which describe protocols hosted on protocols.io. Read more information on sharing protocols at https://plos.org/protocols?utm_medium=editorial-email&utm_source=authorletters&utm_campaign=protocols.

We look forward to receiving your revised manuscript.

Kind regards,

Amirreza Khalaji

Academic Editor

PLOS One

Journal Requirements:

1. If the reviewer comments include a recommendation to cite specific previously published works, please review and evaluate these publications to determine whether they are relevant and should be cited. There is no requirement to cite these works unless the editor has indicated otherwise.

2. Please review your reference list to ensure that it is complete and correct. If you have cited papers that have been retracted, please include the rationale for doing so in the manuscript text, or remove these references and replace them with relevant current references. Any changes to the reference list should be mentioned in the rebuttal letter that accompanies your revised manuscript. If you need to cite a retracted article, indicate the article’s retracted status in the References list and also include a citation and full reference for the retraction notice.

[Note: HTML markup is below. Please do not edit.]

Reviewers' comments:

Reviewer's Responses to Questions

**Comments to the Author**

1. If the authors have adequately addressed your comments raised in a previous round of review and you feel that this manuscript is now acceptable for publication, you may indicate that here to bypass the “Comments to the Author” section, enter your conflict of interest statement in the “Confidential to Editor” section, and submit your "Accept" recommendation.

Reviewer #1: All comments have been addressed

Reviewer #2: All comments have been addressed

Reviewer #3: All comments have been addressed

Reviewer #4: (No Response)

**********

2. Is the manuscript technically sound, and do the data support the conclusions?

The manuscript must describe a technically sound piece of scientific research with data that supports the conclusions. Experiments must have been conducted rigorously, with appropriate controls, replication, and sample sizes. The conclusions must be drawn appropriately based on the data presented.

Reviewer #1: Yes

Reviewer #2: Yes

Reviewer #3: Yes

Reviewer #4: Yes

**********

3. Has the statistical analysis been performed appropriately and rigorously?

Reviewer #1: Yes

Reviewer #2: Yes

Reviewer #3: Yes

Reviewer #4: Yes

**********

4. Have the authors made all data underlying the findings in their manuscript fully available?

The PLOS Data policy requires authors to make all data underlying the findings described in their manuscript fully available without restriction, with rare exception (please refer to the Data Availability Statement in the manuscript PDF file). The data should be provided as part of the manuscript or its supporting information, or deposited to a public repository. For example, in addition to summary statistics, the data points behind means, medians and variance measures should be available. If there are restrictions on publicly sharing data—e.g. participant privacy or use of data from a third party—those must be specified.

Reviewer #1: Yes

Reviewer #2: No

Reviewer #3: Yes

Reviewer #4: Yes

**********

5. Is the manuscript presented in an intelligible fashion and written in standard English?

PLOS ONE does not copyedit accepted manuscripts, so the language in submitted articles must be clear, correct, and unambiguous. Any typographical or grammatical errors should be corrected at revision, so please note any specific errors here.

Reviewer #1: Yes

Reviewer #2: Yes

Reviewer #3: (No Response)

Reviewer #4: Yes

**********

6. Review Comments to the Author

Please use the space provided to explain your answers to the questions above. You may also include additional comments for the author, including concerns about dual publication, research ethics, or publication ethics. (Please upload your review as an attachment if it exceeds 20,000 characters)

Reviewer #1: Thank you for the effort invested in addressing my comments. The paper makes a valuable contribution to the field and meets the journal’s standards, and I therefore recommend its acceptance. One final comment: while the tables indicate that your model outperforms comparable approaches, the illustrative examples (figs 5-7) suggest that some other models achieve better performance in specific cases. You may wish to clarify this discrepancy or select examples from your dataset that more accurately reflect the reported results.

Reviewer #2: (No Response)

Reviewer #3: (No Response)

Reviewer #4: Dear authors,

thank you for considering the raised comments. The current version of your manuscript is significantly enhanced; however, it requires some further adjustments.

• The Methods section contains contradictory statements about how the datasets were split. For example, one passage reads: “we followed the standard practice in the literature by randomly splitting them into training and testing sets at an 8:2 ratio.”. Immediately afterwards, another states: “This study adopts patient-level split to divide all datasets into the training set and the test set.”. These two sentences directly conflict (random image-level splitting vs. patient-level splitting). It is unclear which procedure was actually used. This ambiguity is critical because patient-level splitting (to avoid data leakage) versus random image splitting have very different implications for validity. The authors must clarify and correct this in the Methods so that the text consistently describes the actual experimental procedure.

• It is unclear whether an independent test set or validation set was used. The Methods text discusses a training/test split, but the implementation details say “16 [batch size] for the training set and 8 for the validation set”. Is this “validation set” actually the 20% held-out test set? How was early stopping applied? The manuscript does not explicitly describe how the data were partitioned and used during training versus final evaluation.

• The text contains conflicting information on image resizing. The Dataset section first says “All images were resized to 256 × 256 pixels”. But later, the experimental details state “all images resized to 224 × 224 pixels”. This contradiction should be resolved. The authors should state a single image size and ensure the description of preprocessing is consistent throughout. Inconsistent preprocessing raises concerns about the reliability of reported results.

• The manuscript does not mention whether any data augmentation (rotations, flips, etc.) was applied. Such details are important in medical image segmentation. Similarly, while the response letter mentions an early-stopping strategy, the main text does not describe whether early stopping or how a validation set was used for hyperparameter selection. The Methods should explicitly report any augmentation methods, exact splits (train/val/test), and stopping criteria to ensure reproducibility.

• Tables now include “mean ± standard deviation” for metrics, which addresses the prior comment about variability. However, the text should also explain in the Methods that each experiment was repeated (20 times) with different random seeds, and results averaged. Currently, the text does not describe this, although the tables show it. A brief statement (e.g. “Each model was trained 20 times with different random seeds; performance metrics are reported as mean±SD”) should be added for clarity.

• The Results text states that PM-DUNet “outperforms all comparative methods in core performance metrics of Dice and IoU”. The tables do support that PM-DUNet has the highest Dice and IoU on all three datasets. However, in the Discussion the authors note WRANet has high specificity (Dice) on TN3K but still claim overall superiority. It would help to explicitly acknowledge in the text any exceptions or close competitors in specific metrics. This is not a major flaw, but clarifying which metrics drove the conclusions would improve interpretation.

**********

7. PLOS authors have the option to publish the peer review history of their article (what does this mean?). If published, this will include your full peer review and any attached files.

If you choose “no”, your identity will remain anonymous but your review may still be made public.

**Do you want your identity to be public for this peer review?**  For information about this choice, including consent withdrawal, please see our Privacy Policy.

Reviewer #1: No

Reviewer #2: No

Reviewer #3: No

Reviewer #4: No

**********

[NOTE: If reviewer comments were submitted as an attachment file, they will be attached to this email and accessible via the submission site. Please log into your account, locate the manuscript record, and check for the action link "View Attachments". If this link does not appear, there are no attachment files.]

To ensure your figures meet our technical requirements, please review our figure guidelines: https://journals.plos.org/plosone/s/figures

You may also use PLOS’s free figure tool, NAAS, to help you prepare publication quality figures: https://journals.plos.org/plosone/s/figures#loc-tools-for-figure-preparation.

NAAS will assess whether your figures meet our technical requirements by comparing each figure against our figure specifications.

# Author comment: Author response to Decision Letter 2

**DOI:** 10.1371/journal.pone.0353684.r004

10 Mar 2026

Dear Editor and Reviewers,

We would like to express our sincere gratitude to the editor for giving us the opportunity to revise our manuscript. We are deeply thankful to all reviewers for their constructive, detailed, and valuable comments, which have been of great importance in improving the quality of our manuscript.

Response to Reviewer #4

Comment 1: Contradiction about dataset split (random split vs. patient-level split)

Response: We apologize for the contradictory description in the original manuscript. To avoid data leakage and ensure the validity of experimental results, we strictly adopted patient-level splitting for all datasets in our experiments. We have revised the corresponding sentence in the manuscript. All inconsistent descriptions have been unified and corrected to ensure the experimental procedure is clearly and accurately reported.

Comment 2: Unclear validation set / independent test set / early stopping

Response: We sincerely apologize for the unclear description. We clarify as follows:

The dataset was partitioned into a training set (80%) and a test set (20%) at the patient level. Early stopping was adopted based on the loss on the validation set, with a patience of 15 epochs.

Comment 3: Contradiction on image resizing (256×256 vs. 224×224)

Response: We apologize for this error. All images in our experiments were uniformly resized to 224 × 224 pixels throughout the preprocessing. We have deleted the incorrect description of “256 × 256” and ensured that all parts of the manuscript are consistent with 224 × 224 pixels.

Comment 4: Missing data augmentation

Response: We have added the missing experimental details in the Materials and methods section: We don’t applied any data augmentation during training.

Comment 5: Missing description of repeated experiments (20 times with random seeds)

Response: We have added the following description in the manuscript:

Each experiment was repeated 20 times with different random seeds, and the performance metrics are reported as the mean ± standard deviation.

This ensures consistency between the text and the tables.

Comment 6: Acknowledge that WRANet has higher specificity (SE) on TN3K

Response: We agree with the reviewer. We have revised the Discussion section to acknowledge that. This makes the conclusion more objective and rigorous.

Thank you again for your valuable comments.

Attachment Submitted filename: Response.docx

# Peer review: Decision Letter 2

**DOI:** 10.1371/journal.pone.0353684.r005

**Authors:** Yongjie Li

24 Apr 2026

PONE-D-25-53692R2

PM-DUnet:Fusing Long-Range Dependencies and Attention in a Dual-U Architecture for Thyroid Nodule Segmentation

PLOS One

Dear Dr. Cheng,

Thank you for submitting your manuscript to PLOS ONE. After careful consideration, we feel that it has merit but does not fully meet PLOS ONE’s publication criteria as it currently stands. Therefore, we invite you to submit a revised version of the manuscript that addresses the points raised during the review process.

==============================

The credibility and reproducibility of the reported results should be further improved.

==============================

Please submit your revised manuscript by Jun 08 2026 11:59PM. If you will need more time than this to complete your revisions, please reply to this message or contact the journal office at plosone@plos.org. When you're ready to submit your revision, log on to https://www.editorialmanager.com/pone/ and select the 'Submissions Needing Revision' folder to locate your manuscript file.

Please include the following items when submitting your revised manuscript:

- A letter that responds to each point raised by the academic editor and reviewer(s). You should upload this letter as a separate file labeled 'Response to Reviewers'.
- A marked-up copy of your manuscript that highlights changes made to the original version. You should upload this as a separate file labeled 'Revised Manuscript with Track Changes'.
- An unmarked version of your revised paper without tracked changes. You should upload this as a separate file labeled 'Manuscript'.

If you would like to make changes to your financial disclosure, please include your updated statement in your cover letter. Guidelines for resubmitting your figure files are available below the reviewer comments at the end of this letter.

If applicable, we recommend that you deposit your laboratory protocols in protocols.io to enhance the reproducibility of your results. Protocols.io assigns your protocol its own identifier (DOI) so that it can be cited independently in the future. For instructions see: https://journals.plos.org/plosone/s/submission-guidelines#loc-laboratory-protocols. Additionally, PLOS ONE offers an option for publishing peer-reviewed Lab Protocol articles, which describe protocols hosted on protocols.io. Read more information on sharing protocols at https://plos.org/protocols?utm_medium=editorial-email&utm_source=authorletters&utm_campaign=protocols.

As the corresponding author, your ORCID iD is verified in the submission system and will appear in the published article. PLOS supports the use of ORCID, and we encourage all coauthors to register for an ORCID iD and use it as well. Please encourage your coauthors to verify their ORCID iD within the submission system before final acceptance, as unverified ORCID iDs will not appear in the published article. *Only*  the individual author can complete the verification step; PLOS staff *cannot*  verify ORCID iDs on behalf of authors.

We look forward to receiving your revised manuscript.

Kind regards,

Yongjie Li

Academic Editor

PLOS One

Journal Requirements:

1. If the reviewer comments include a recommendation to cite specific previously published works, please review and evaluate these publications to determine whether they are relevant and should be cited. There is no requirement to cite these works unless the editor has indicated otherwise.

2. Please review your reference list to ensure that it is complete and correct. If you have cited papers that have been retracted, please include the rationale for doing so in the manuscript text, or remove these references and replace them with relevant current references. Any changes to the reference list should be mentioned in the rebuttal letter that accompanies your revised manuscript. If you need to cite a retracted article, indicate the article’s retracted status in the References list and also include a citation and full reference for the retraction notice.

Additional Editor Comments:

The credibility and reproducibility of the reported results should be improved.

[Note: HTML markup is below. Please do not edit.]

Reviewers' comments:

Reviewer's Responses to Questions

**Comments to the Author**

1. If the authors have adequately addressed your comments raised in a previous round of review and you feel that this manuscript is now acceptable for publication, you may indicate that here to bypass the “Comments to the Author” section, enter your conflict of interest statement in the “Confidential to Editor” section, and submit your "Accept" recommendation.

Reviewer #2: All comments have been addressed

Reviewer #4: (No Response)

**********

2. Is the manuscript technically sound, and do the data support the conclusions?

The manuscript must describe a technically sound piece of scientific research with data that supports the conclusions. Experiments must have been conducted rigorously, with appropriate controls, replication, and sample sizes. The conclusions must be drawn appropriately based on the data presented.

Reviewer #2: Yes

Reviewer #4: Partly

**********

3. Has the statistical analysis been performed appropriately and rigorously?

Reviewer #2: Yes

Reviewer #4: Yes

**********

4. Have the authors made all data underlying the findings in their manuscript fully available?

The PLOS Data policy requires authors to make all data underlying the findings described in their manuscript fully available without restriction, with rare exception (please refer to the Data Availability Statement in the manuscript PDF file). The data should be provided as part of the manuscript or its supporting information, or deposited to a public repository. For example, in addition to summary statistics, the data points behind means, medians and variance measures should be available. If there are restrictions on publicly sharing data—e.g. participant privacy or use of data from a third party—those must be specified.

Reviewer #2: Yes

Reviewer #4: Yes

**********

5. Is the manuscript presented in an intelligible fashion and written in standard English?

PLOS ONE does not copyedit accepted manuscripts, so the language in submitted articles must be clear, correct, and unambiguous. Any typographical or grammatical errors should be corrected at revision, so please note any specific errors here.

Reviewer #2: Yes

Reviewer #4: Yes

**********

6. Review Comments to the Author

Please use the space provided to explain your answers to the questions above. You may also include additional comments for the author, including concerns about dual publication, research ethics, or publication ethics. (Please upload your review as an attachment if it exceeds 20,000 characters)

Reviewer #2: All comments raised during the review process have been thoroughly addressed, and the manuscript has been revised accordingly. I believe that the revised version satisfactorily resolves all concerns and that no outstanding issues remain.

Reviewer #4: I reviewed the manuscript “PM-DUnet: Fusing Long-Range Dependencies and Attention in a Dual-U Architecture for Thyroid Nodule Segmentation”. Based on the current version, I see some internal inconsistencies through the methods.

1. The training/validation/test protocol is contradictory and not reproducible.

The response-to-reviewers section states: “The dataset was partitioned into a training set (80%) and a test set (20%) at the patient level. Early stopping was adopted based on the loss on the validation set, with a patience of 15 epochs.” However, the Results section states: “We used an early stopping mechanism, which stopped training the model after 20 consecutive rounds when all metrics had decreased.” These statements cannot both be true as written. One version implies a validation-loss criterion with patience 15; the other implies a metric-based stopping rule with patience 20. If I catch it wrongly please clarify this issue.

2. The method section states: “Each subsequence xk is then processed independently by a weight-shared Mamba block.” Later, when discussing the 8-path ablation, the manuscript explains the performance drop as follows: “This may be attributed to excessive parameter fragmentation, where each Mamba block has too few channels to learn meaningful representations.” If the path blocks are truly weight-shared, then the explanation based on fragmentation of each Mamba block would not well be justified as written. The manuscript must clarify whether the parallel Mamba paths use shared or separate parameters, and the authors should provide parameter counts for the 2-, 4-, and 8-path variants.

3. In Figure 4 and its caption state: “The module processes features through MaxPath, AvgPath, and a shared MLP layer.” However, the mathematical description defines the attention map as “Ms = σ(f7×7([AvgPoolc(X); MaxPoolc(X)])),” which is a convolution-based spatial attention mechanism rather than an MLP-based one. Please clarify this.

4. The manuscript states: “TG3K Dataset: [21] … comprising 3478 ultrasound images from 3478 patients” and “TN3K Dataset: [22] This dataset consists of 3108 thyroid ultrasound images from 3108 patients.” Yet published descriptions of TG3K describe it as a dataset extracted from 16 ultrasound videos, with about 3,585 images, not 3,478 images from 3,478 patients. Published descriptions of TN3K describe it as an open-access thyroid nodule dataset containing 3,493 images from 2,421 patients, not 3,108 images from 3,108 patients. Please reassure that the mentioned numbers and samples in the manuscript to be correct.

5. The main superiority claim is overstated relative to the manuscript’s own evidence.

The manuscript claims in the abstract that “Results show PM-DUNet outperforms state-of-the-art methods on key metrics,” and later concludes that PM-DUNet “outperforms existing state-of-the-art segmentation methods across various performance metrics.” Yet the manuscript itself acknowledges that “WRANet achieves a slightly higher Sensitivity (SE) score on the TN3K dataset compared with our PM-DUNet.” Given the unresolved contradictions, the current superiority claim is too strong. It should be moderated until the experimental record is internally consistent.

**********

7. PLOS authors have the option to publish the peer review history of their article (what does this mean?). If published, this will include your full peer review and any attached files.

If you choose “no”, your identity will remain anonymous but your review may still be made public.

**Do you want your identity to be public for this peer review?**  For information about this choice, including consent withdrawal, please see our Privacy Policy.

Reviewer #2: No

Reviewer #4: No

**********

[NOTE: If reviewer comments were submitted as an attachment file, they will be attached to this email and accessible via the submission site. Please log into your account, locate the manuscript record, and check for the action link "View Attachments". If this link does not appear, there are no attachment files.]

To ensure your figures meet our technical requirements, please review our figure guidelines: https://journals.plos.org/plosone/s/figures

You may also use PLOS’s free figure tool, NAAS, to help you prepare publication quality figures: https://journals.plos.org/plosone/s/figures#loc-tools-for-figure-preparation.

NAAS will assess whether your figures meet our technical requirements by comparing each figure against our figure specifications.

# Author comment: Author response to Decision Letter 3

**DOI:** 10.1371/journal.pone.0353684.r006

22 Jun 2026

Response to Reviewer #4

Point 1: The training/validation/test protocol is contradictory and not reproducible...

Response: We sincerely apologize for the confusion caused by the contradictory statements regarding our training protocol. We confirm that the dataset was partitioned at the patient level into an 80/20 split for training and testing, respectively. Early stopping was indeed implemented based on the validation loss with a patience of 15 epochs. We have corrected the Results section in the manuscript to accurately reflect this protocol and have removed any ambiguous phrasing.

Point 2: The method section states: “Each subsequence xk is then processed independently by a weight-shared Mamba block.” Later... explains the performance drop as follows: “attributed to excessive parameter fragmentation”...

Response: We appreciate your careful reading and constructive feedback. because the parallel paths share parameters, the overall parameter count per block does not decrease. Rather, dividing the feature map into 8 paths reduces the channel dimension processed by the shared block to C/8. This drastically limits the block's representation capacity, leaving it with too few channels to learn robust and meaningful feature relationships. We have revised the Ablation Study section to accurately explain this dynamic, and we have included the exact parameter counts for the 2-, 4-, and 8-path variants to provide complete transparency.

Point 3: In Figure 4 and its caption state: “The module processes features through MaxPath, AvgPath, and a shared MLP layer.” However, the mathematical description defines the attention map as... a convolution-based spatial attention mechanism...

Response: Thank you for pointing out this inconsistency. The mathematical description outlining a 7x7 convolutional layer is correct. The mention of a "shared MLP" in the figure caption was an oversight carried over from a previous draft of the architecture. We have updated Figure 4's caption to correctly state that the module utilizes a convolutional layer.

Point 4: The manuscript states: “TG3K Dataset: ... comprising 3478 ultrasound images from 3478 patients” and “TN3K Dataset: ... 3108 thyroid ultrasound images from 3108 patients.” Yet published descriptions...

Response: We apologize for the inaccuracies regarding the dataset statistics. We have double-checked the numbers with the original dataset publications and corrected the manuscript. The TG3K dataset description now correctly states it contains 3,583 images extracted from video sequences, and the TN3K description has been corrected to 3,493 images from 2,421 patients.

Point 5: The main superiority claim is overstated relative to the manuscript’s own evidence...

Response: We agree that our previous wording was slightly overstated, particularly given that WRANet achieves a higher Sensitivity score on the TN3K dataset. We have revised the Abstract and Conclusion to moderate these claims. The text now states that our method achieves highly competitive performance and outperforms state-of-the-art methods on most key metrics (such as Dice and IoU), ensuring our claims align accurately with the experimental evidence.

Attachment Submitted filename: Reponse.docx

# Editor report: Decision Letter 3

**DOI:** 10.1371/journal.pone.0353684.r007

**Authors:** Yongjie Li

29 Jun 2026

PM-DUnet:Fusing Long-Range Dependencies and Attention in a Dual-U Architecture for Thyroid Nodule Segmentation

PONE-D-25-53692R3

Dear Dr. Cheng,

We’re pleased to inform you that your manuscript has been judged scientifically suitable for publication and will be formally accepted for publication once it meets all outstanding technical requirements.

Within one week, you’ll receive an e-mail detailing the required amendments. When these have been addressed, you’ll receive a formal acceptance letter and your manuscript will be scheduled for publication.

An invoice will be generated when your article is formally accepted. Please note, if your institution has a publishing partnership with PLOS and your article meets the relevant criteria, all or part of your publication costs will be covered. Please make sure your user information is up-to-date by logging into Editorial Manager at Editorial Manager® and clicking the ‘Update My Information' link at the top of the page. For questions related to billing, please contact billing support.

If your institution or institutions have a press office, please notify them about your upcoming paper to help maximize its impact. If they’ll be preparing press materials, please inform our press team as soon as possible -- no later than 48 hours after receiving the formal acceptance. Your manuscript will remain under strict press embargo until 2 pm Eastern Time on the date of publication. For more information, please contact onepress@plos.org.

Kind regards,

Yongjie Li

Academic Editor

PLOS One

Additional Editor Comments (optional):

The concerns from Reviewer #4 have been well addressed by the authors.

Reviewers' comments:

# Editor report: Acceptance letter

**DOI:** 10.1371/journal.pone.0353684.r008

**Authors:** Yongjie Li

PONE-D-25-53692R3

PLOS One

Dear Dr. Cheng,

I'm pleased to inform you that your manuscript has been deemed suitable for publication in PLOS One. Congratulations! Your manuscript is now being handed over to our production team.

At this stage, our production department will prepare your paper for publication. This includes ensuring the following:

* All references, tables, and figures are properly cited

* All relevant supporting information is included in the manuscript submission,

* There are no issues that prevent the paper from being properly typeset

You will receive further instructions from the production team, including instructions on how to review your proof when it is ready. Please keep in mind that we are working through a large volume of accepted articles, so please give us a few days to review your paper and let you know the next and final steps.

Lastly, if your institution or institutions have a press office, please let them know about your upcoming paper now to help maximize its impact. If they'll be preparing press materials, please inform our press team within the next 48 hours. Your manuscript will remain under strict press embargo until 2 pm Eastern Time on the date of publication. For more information, please contact onepress@plos.org.

You will receive an invoice from PLOS for your publication fee after your manuscript has reached the completed accept phase. If you receive an email requesting payment before acceptance or for any other service, this may be a phishing scheme. Learn how to identify phishing emails and protect your accounts at https://explore.plos.org/phishing.

If we can help with anything else, please email us at customercare@plos.org.

Thank you for submitting your work to PLOS ONE and supporting open access.

Kind regards,

PLOS ONE Editorial Office Staff

on behalf of

Professor Yongjie Li

Academic Editor

PLOS One
